# Promoting Autophagy Mitigates Stress‐Induced Remodeling in Patient iPSC‐CMs with the Phospholamban R9C Mutation

**DOI:** 10.1002/advs.202511480

**Published:** 2025-11-27

**Authors:** Qi Yu, Yawei Shen, Robert J. Barndt, Karim Sallam, Ying Tang, Cameron E. Brown, Xiao Li, Stephen Y. Chan, Joseph C. Wu, Qing Liu, Haodi Wu

**Affiliations:** ^1^ Pittsburgh Heart, Lung, and Blood Vascular Medicine Institute University of Pittsburgh School of Medicine Pittsburgh PA 15261 USA; ^2^ Department of Biological Sciences Clemson University Clemson SC 29634 USA; ^3^ Center for Human Genetics Clemson University Greenwood SC 29646 USA; ^4^ College of Fisheries Henan Normal University Xinxiang Henan 453007 China; ^5^ Stanford Cardiovascular Institute Department of Medicine Division of Cardiovascular Medicine and Institute for Stem Cell Biology and Regenerative Medicine Stanford University School of Medicine Stanford CA 94304 USA; ^6^ Division of Cardiology Department of Medicine University of Pittsburgh School of Medicine Pittsburgh PA 15261 USA

**Keywords:** autophagy, dilated cardiomyopathy, genome editing, induced pluripotent stem cell model, mutation, phospholamban

## Abstract

Phospholamban (PLN) is a key regulator of adrenergic signaling and calcium homeostasis in cardiomyocytes. The PLN R9C mutation causes early‐onset dilated cardiomyopathy (DCM) and premature death, yet the mechanisms underlying its pathogenic remodeling remain unclear. In this study, patient‐specific induced pluripotent stem cell‐derived cardiomyocytes (iPSC‐CMs) harboring the PLN R9C and their isogenic corrected controls are generated to investigate the molecular and functional consequences of the mutation. At baseline, PLN R9C iPSC‐CMs exhibit near‐normal calcium handling but blunt β‐adrenergic signaling and slightly enhanced contractility. Under functional stress induced by a pro‐maturation medium, mutant cells develop marked sarcomere disarray, impaired calcium handling, elevated diastolic calcium, and reduced contractile force, whereas corrected cells show adaptive improvement. Transcriptomic and biochemical analyses reveal activation of proteostasis pathways but accumulation of PLN pentamers and impaired autophagic flux, suggesting that autophagic overload contributes to functional remodeling. Treatment with the autophagy activator metformin mitigates sarcomere disorganization, restores calcium homeostasis, and improves contractility in patient‐derived iPSC‐CMs. These findings are further validated in wild‐type and genome‐engineered PLN R9C iPSC‐CMs. Collectively, the study demonstrates that PLN R9C drives stress‐induced pathological remodeling by disrupting proteostasis, and that enhancing autophagic flux offers a promising therapeutic strategy for DCM patients carrying PLN mutations.

## Graphical abstract

The Phospholamban (PLN) R9C mutation reduces SERCA2a binding, increasing calcium recycling and baseline contractility. However, the excess of free PLN promotes pentamer formation, limiting phosphorylation and blunting β‐adrenergic signaling. Under cardiac stress, enhanced functional demands overwhelm proteostasis in PLN R9C cells, leading to misfolded protein accumulation, sarcomere disarray, and functional remodeling, defects that were partially rescued by enhancing autophagy.

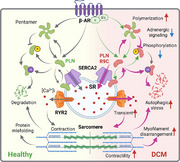

## 1 Introduction

Cardiac muscle contraction and relaxation are finely tuned by intracellular calcium homeostasis. During systole, membrane depolarization activates L‐type calcium channels (LCCs), permitting calcium influx into the cytosol. This triggers calcium‐induced calcium release (CICR) via ryanodine receptors (RyR2), releasing substantial calcium from the sarcoplasmic reticulum (SR).^[^ ^1^ ^]^ This free calcium binds the troponin complex within the sarcomeric contractile apparatus, initiating contraction. During diastole, the sarco/endoplasmic reticulum Ca^2^⁺‐ATPase (SERCA) recycles calcium into the SR, a process regulated by Phospholamban (PLN), a small, single‐pass membrane protein that inhibits SERCA's calcium affinity.^[^ ^2^ ^]^ β‐Adrenergic signaling in the heart phosphorylates PLN at Ser16 (by cAMP‐dependent protein kinase A, PKA) and Thr17 (by calcium/calmodulin‐dependent protein kinase II, CaMKII), inducing a conformational shift that relieves SERCA inhibition.^[^ ^3^ ^]^ The enhancement of SERCA activity facilitates accelerated calcium reuptake into the SR, thereby increasing calcium recycling (lusitropy), heart rate (chronotropy), and contractility (inotropy), thus releasing the reserved cardiac pumping power in response to higher functional demand. Therefore, PLN is a key regulatory molecule that connects calcium handling and β‐adrenergic signaling pathways in the heart.^[^ ^4^ ^]^

Phospholamban (PLN) mutations disrupt its regulation of cardiac function. Several PLN mutations, including R14del, R9C, R25C, L39X, E2X, and others, have been identified in families with dilated cardiomyopathy (DCM), a common disorder that often progresses to heart failure (HF).^[^ ^5^, ^6^, ^7^, ^8^, ^9^, ^10^, ^11^ ^]^ Among them, R14del, R9C, and R25C are the most prevalent ones in DCM. Studies have demonstrated that PLN‐R14del mutation results in impaired contractile function, increased myocardial fibrosis, PLN protein aggregation, and increased risk of ventricular tachycardia (VT).^[^ ^12^, ^13^, ^14^ ^]^ The PLN‐R25C mutation was shown to cause the super‐inhibition of SERCA2a, decreased SR calcium load, increased SR calcium leakage and CaMKII activity, which result in weaker contraction and arrhythmia.^[^ ^7^, ^15^ ^]^ The PLN‐R9C mutation was first identified in patients with early‐onset DCM by Schmitt et al., which may bind irreversibly to the catalytic subunit of PKA (PKA‐C), thus preventing phosphorylation of PLN at Ser16.^[^ ^9^ ^]^ This was further confirmed by defective β‐adrenergic signaling in the heart of PLN‐R9C animal models.^[^ ^16^, ^17^ ^]^ Additional studies also indicate PLN R9C mutation affects the PLN's binding affinity to SERCA and disrupts the monomer‐pentamer equilibrium of PLN protein.^[^ ^18^, ^19^ ^]^ However, the role of PLN R9C in human DCM remains unclear due to limited human sample‐based studies, and there is no specific treatment for DCM patients with this mutation to date.

To better understand the pathological role of PLN R9C mutation in human cardiomyocytes, the current study generated induced pluripotent stem cells (iPSCs) disease models with DCM patient‐specific lines (PA) and CRISPR/Cas9 corrected isogenic lines (Corr). In our observation, patient iPSC‐derived cardiomyocytes (iPSC‐CMs) exhibited blunted β‐adrenergic signaling as reported, while maintaining near normal calcium handling and contractility.^[^ ^20^ ^]^ Upon treatment with maturation medium (MM), which enhances metabolic and functional maturation, PA iPSC‐CMs exhibited functional deficits, while corrected cells showed improved functional performance. This suggests that increased functional demand triggers pathological remodeling in PLN R9C mutation‐harboring cells. Transcriptomic and signaling analysis revealed that MM treatment exacerbated autophagic stress, protein aggregation, sarcomere disarray, and functional decline in PA cells. Finally, pharmacological activation of autophagic signaling pathways partially restores structural and functional integrity in PA iPSC‐CMs under stress, confirming proteostasis dysregulation as a key pathological mechanism of PLN R9C mutation. These findings were validated in wild‐type (WT) and the PLN R9C mutation knock‐in (GE) iPSC‐CMs. In summary, this study provides novel mechanistic insights and potential therapeutic targets for PLN R9C mutation‐related DCM.

## 2 Results

### 2.1 Patient‐Derived iPSC‐CMs with PLN R9C Mutation Exhibited Blunted β‐Adrenergic Signaling

DCM (PA, patient‐specific cells) iPSC lines were validated for clone formation, pluripotency markers, and in vitro differentiation (Figure S1A–C, Supporting Information), with genotyping confirming the mutation. DCM patient‐ lines were differentiated into beating cardiomyocytes using a standard protocol.^[^ ^21^ ^]^ The fluorescence‐activated cell sorting (FACS) of cardiac troponin T (TNNT2)‐positive cells showed that the PLN R9C mutation did not affect differentiation efficiency or purity (Figure S1D, Supporting Information). Isogenic control iPSC lines (Corr, PLN R9C mutation corrected cells) were generated by correcting the PLN C25T (p.R9C) mutation in the DCM iPSC lines to wild‐type using CRISPR (Figure S1E,F, Supporting Information).^[^ ^22^ ^]^ The pluripotency of the Corr iPSCs, as well as the specificity of the sgRNA‐induced mutation correction, were further validated (Figure S1G–J, Supporting Information). Structural and functional analyses of PA and Corr iPSC‐CMs were performed with purified iPSC‐CMs >35 days postdifferentiation.

**Figure 1:**
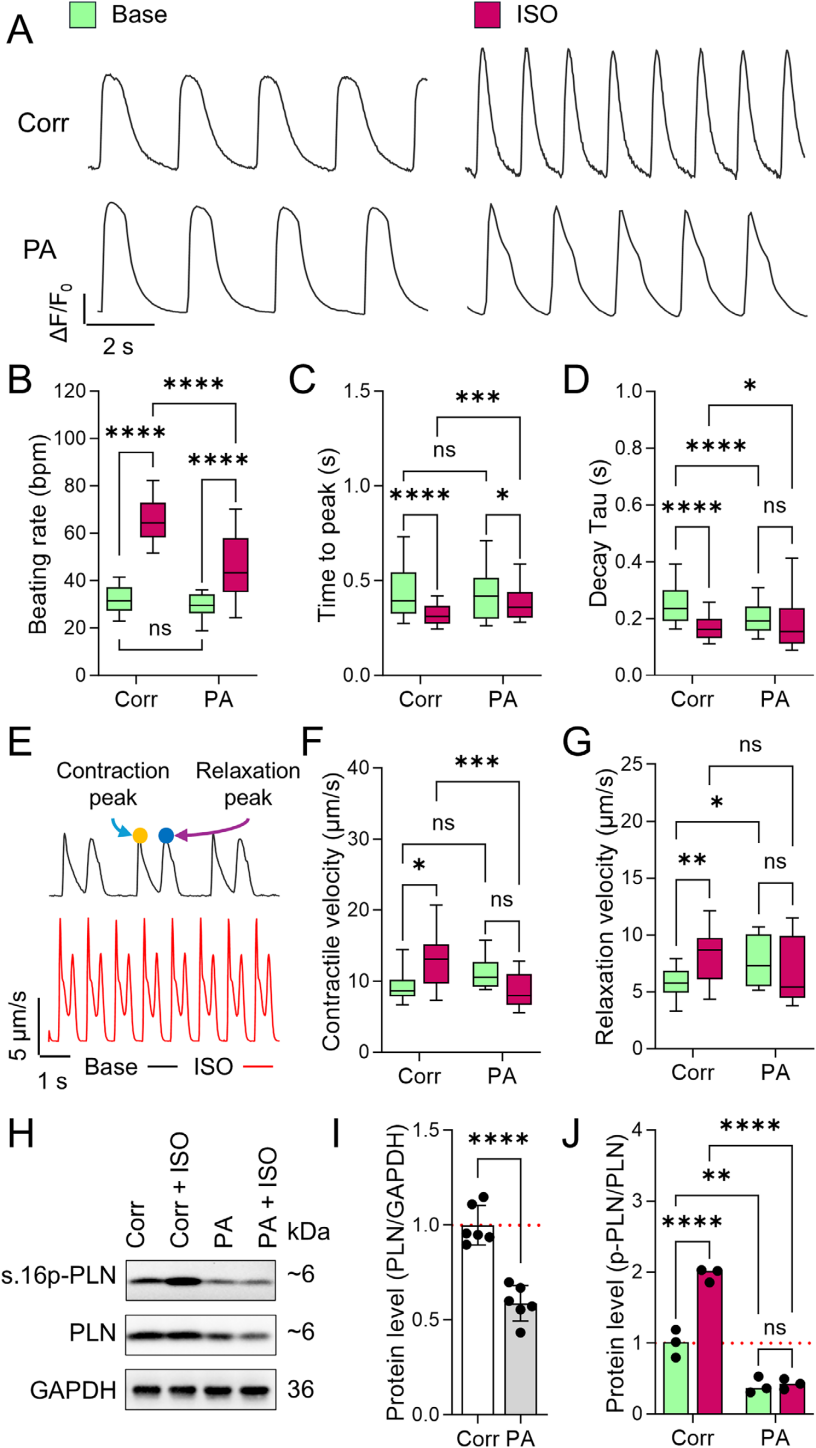
Impaired adrenergic signaling in patient iPSC‐CMs with PLN R9C mutation. A) Representative Fluo‐4 AM calcium transient trace in Corr and PA iPSC‐CMs before and after isoproterenol (ISO) treatment. B−D) Quantification of calcium handling parameters: beating rate B), and transient decay Tau C), and transient rise time D). *N* = 180, 131, 242, and 108 cells of Corr, PA, Corr ISO, and PA ISO groups from at least 3 independent experiments. E) Representative traction force microscopy (TFM) traces of the displacement velocity of beating iPSC‐CMs before and after ISO treatment. Arrows indicate the contraction peak (yellow dot) and relaxation peak (blue dot) in a beating episode. F,G) Traction force microscopy (TFM) measurement of the peak contraction F) and relaxation velocities G) of iPSC‐CMs in monolayers. *N* = 28, 27, 29, and 42 ROIs of Corr, PA, Corr ISO, and PA ISO groups from at least 3 independent experiments. NS, no significance, *p < 0.05, **p < 0.01, ***p < 0.001, and ****p < 0.0001 by 2‐way ANOVA test followed by Holm–Sidak method. H) Quantification of the PLN and phosphorylation form of PLN at Ser16 before and after acute ISO treatment by Western blot. I) The expression of PLN was significantly decreased in PA iPSC‐CMs compared to Corr cells. Results from 6 independent experiments. ****p < 0.0001 by students’ *t*‐test. J) ISO treatment significantly increased the phosphorylation of PLN (measured as the ratio of Ser16‐PLN/PLN abundance) in Corr but not PA iPSC‐CMs. Results from 3 independent experiments. **p < 0.01, and ****p < 0.0001 by 2‐way ANOVA test followed by Holm–Sidak method.

Functional assessment of PA and Corr iPSC‐CMs via Fluo‐4 AM calcium imaging revealed comparable baseline calcium handling (beating rate, transient time‐to‐peak; **Figure** **1**A–C), though PA cells showed faster calcium recycling (shorter decay Tau; Figure 1D). Traction force microscopy (TFM) indicated no difference in contractile velocity, but relaxation velocity was slightly elevated in PA cells (Figure 1E–G). Following β‐adrenergic stimulation by 100 nm isoproterenol (ISO) treatment, Corr iPSC‐CMs exhibited accelerated beating rate, calcium handling, and contraction/relaxation velocities (Figure 1B–G), whereas PA cells showed a blunted response, in terms of modest increases in beating rate and calcium transient elevation, and unchanged decay Tau and contractility (Figure 1B–G). This suggests impaired β‐adrenergic signaling in PA iPSC‐CMs. Western blot analysis revealed reduced total PLN level and significantly lower Ser16 phosphorylation in PA cells, both at baseline and post‐ISO treatment, likely due to the increased instability of mutant PLN and its resistance to PKA phosphorylation (Figure 1H–J).^[^ ^23^ ^]^

### 2.2 In Vitro Maturation Exacerbated the Functional Deficits in Patient iPSC‐CMs

**Figure 2:**
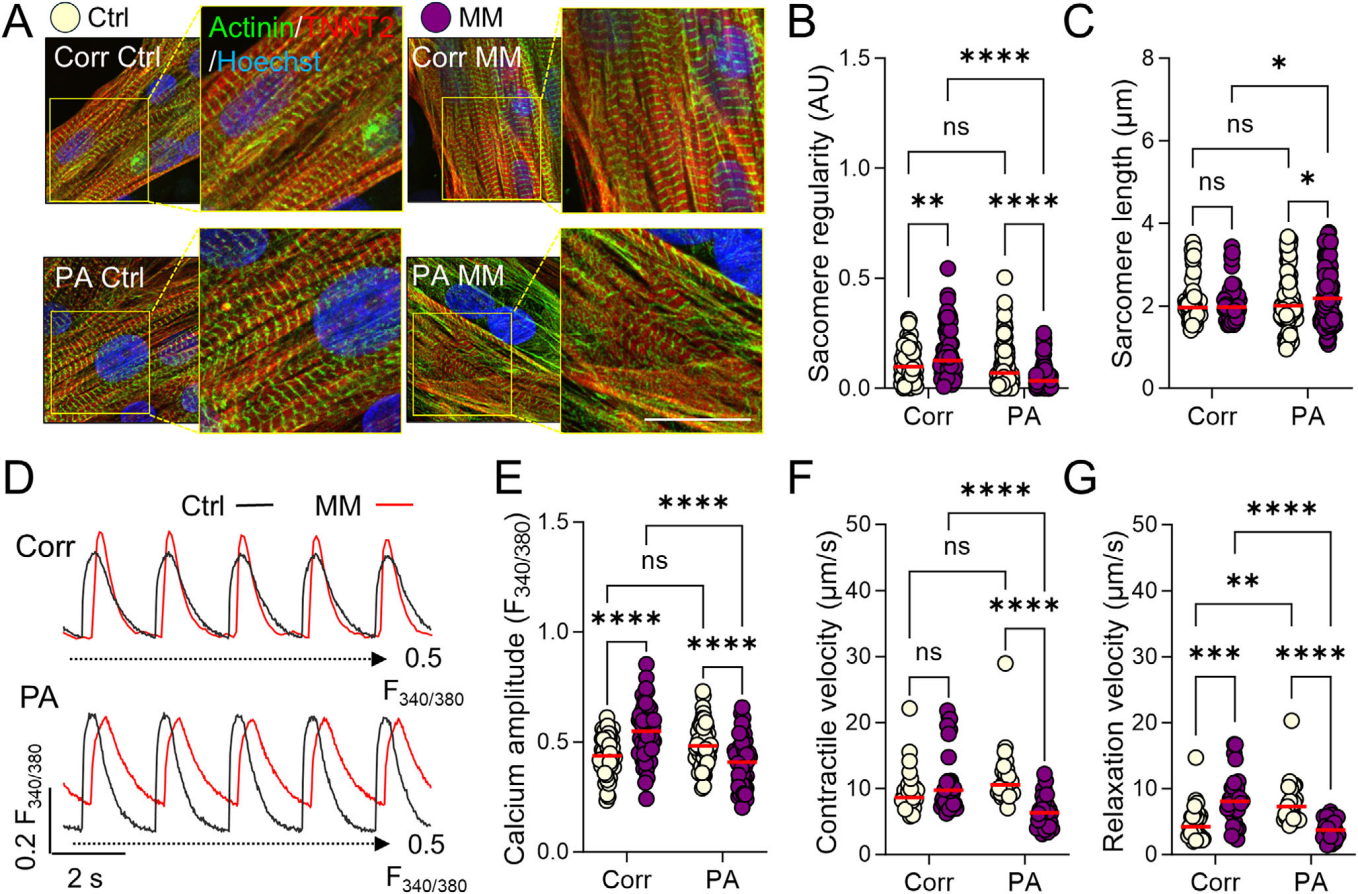
Promaturation medium exaggerated the functional remodeling of patient iPSC‐CMs. A) Immunofluorescence staining of Corr and PA iPSC‐CMs before and after long‐term MM treatment with sarcomere markers (green for α‐actinin and red for TNNT2). B,C) Fast Fourier transformation (FFT) analysis of the sarcomere arrangement regularity B) and sarcomere length C) based on α‐actinin signal distribution in the iPSC‐CMs. *N* = 82, 174, 79, and 134 ROIs of Corr, PA, Corr MM, and PA MM groups from at least 3 independent experiments. D) Representative Fura‐2 AM calcium transient trace in Corr and PA iPSC‐CMs before (black line) and after MM treatment (red line). Black dashed line indicates the F340/F380 ratio at 0.5. E) Comparison of calcium transient amplitude in Fura‐2 AM traces. *N* = 58, 67, 64, and 56 cells of Corr, PA, Corr MM, and PA MM groups from at least 3 independent experiments. F,G) Traction force microscopy (TFM) measurement of peak contractile and relaxation velocity in monolayer Corr and PA iPSC‐CMs before and after MM treatment. *N* = 28, 32, 29, and 30 ROIs of Corr, PA, Corr ISO, and PA ISO groups from at least 3 independent experiments. NS, no significance, *p < 0.05, **p < 0.01, ***p < 0.001, and ****p < 0.0001 by 2‐way ANOVA test followed by Holm–Sidak method.

Acute overexpression of PLN R9C in murine heart has been reported to disrupt PLN‐SERCA2a interactions and promote PLN oligomerization, a process further increased by oxidative stresses.^[^ ^18^ ^]^ Studies also showed mice carrying the PLN R9C transgene alongside two, one, or zero endogenous PLN alleles all developed DCM at varying ages. All these studies suggest the PLN R9C interferes with normal regulation of WT PLN by SERCA2a, contributing to cardiac functional remodeling, which can be exacerbated by pathological stresses.^[^ ^16^ ^]^ However, the regulatory role of PLN R9C under pathological conditions had not been examined in human models. To address this, we sought to determine how functional stresses influence the performance of Corr and PA iPSC‐CMs. We treated Corr and PA iPSC‐CMs at day 35 of differentiation with a pro‐maturation medium (MM) for 7 days to induce both maturation and functional stress, using cells maintained in standard RPMI medium as baseline controls.^[^ ^24^ ^]^ Our prior work has shown that MM promotes iPSC‐CM maturation while simultaneously enhancing challenges to metabolism, protein homeostasis, and autophagic functions, which can exacerbate disease phenotypes in mutant cells.^[^ ^25^ ^]^ After treatment, the structural integrity of the cells was first assessed by immunostaining for sarcomere proteins (**Figure** **2**A). We analyzed sarcomere arrangement using a fast Fourier transform (FFT)‐based algorithm, calculating the regularity of protein distribution as “power” and the average sarcomere length as “period” (Figure S2A,B, Supporting Information). At baseline, both Corr and PA cells exhibited similar power and period values for α‐actinin distribution (Figure 2B,C). After MM treatment, Corr cells showed a modest increase in sarcomere regularity, consistent with enhanced maturation, whereas PA cells displayed marked sarcomere disarray, evidenced by significantly reduced power and an increased sarcomere period (Figure 2B,C). This indicates substantial structural remodeling in patient cells under functional stress.

Functional performance was evaluated next. Calcium handling in MM‐treated Corr cells revealed increased transient amplitude, faster calcium release and recycling rates, and stable diastolic calcium levels and beating rates (Figure 2D,E; and Figure S2C–F, Supporting Information). Traction force microscopy (TFM) further confirmed enhanced contractile and relaxation velocities in these cells (Figure 2F,G). In contrast, MM‐treated PA cells exhibited reduced calcium transient amplitude, elevated diastolic calcium, slower calcium recycling, and decreased contractile and relaxation velocities (Figure 2D–G; and Figure S2C–F, Supporting Information). These findings demonstrate that functional stress via in vitro maturation markedly worsens the structural and functional deficits in PLN R9C iPSC‐CMs compared to their corrected counterparts.

### 2.3 Transcriptome Analysis Revealed Distinct Signaling Pathway Remodeling in Ctrl and DCM iPSC‐CMs under Functional Stresses

**Figure 3:**
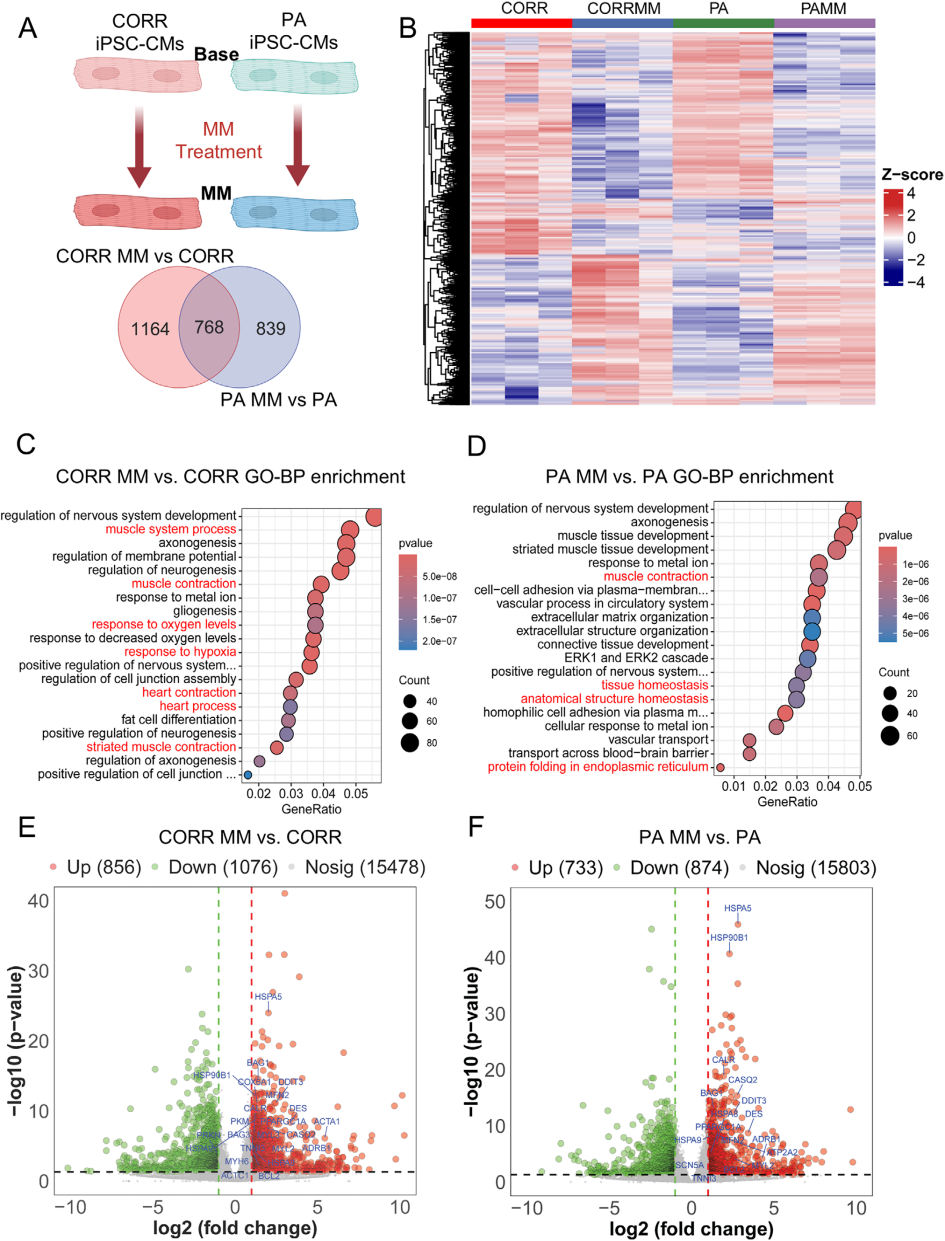
Transcriptome revealed distinct signaling pathways in PA and Corr iPSC‐CMs under functional stresses. A) Number of shared and distinct DEGs in Corr MM versus Base and PA MM versus Base comparisons. B) Heatmap indicates the overall DEG expression pattern of all 4 groups of samples: Corr Base, Corr MM, PA Base, and PA MM. C and D. GO analysis highlighted significantly enriched signaling pathways in Corr and PA cells in response to MM long‐term treatment. E,F) Volcano plot showed the top altered genes after MM treatment in both Corr and PA cells. Some representative genes are highlighted in blue in the plot.

To better understand the underlying mechanism of the maturation challenge‐induced functional deficiency in DCM iPSC‐CMs, we performed genome‐wide transcriptomic analysis of the PA and Corr iPSC‐CMs with and without MM treatment (**Figure** **3**A; and Table S3, Supporting Information). Principal component analysis (PCA) and inter‐sample correlation revealed similar expression patterns and clustering in replicate samples (Figure S3A,B, Supporting Information). We found MM induced 839 and 1164 differentially expressed genes (DEGs) in PA and Corr iPSC‐CMs, and only 768 DEGs were shared between the 2 groups, suggesting distinct regulatory responses to stress (Figure 3A). Clustering heatmaps of DEG expression profiles revealed marked differences in transcriptional responses to stress between PA and Corr iPSC‐CMs (Figure 3B). Moreover, enrichment map profiling of GO biological process terms of the DEGs (*p* value<0.05) revealed that MM treatment activates cardiac maturation‐associated pathways in Corr cells, such as “muscle contraction,” “heart contraction,” “heart process,” and “response to oxygen level.” However, the top enriched GO terms in MM‐treated PA cells are “tissue homeostasis,” “anatomical structure organization,” and “protein folding in endoplasmic reticulum” (Figure 3C,D). Further analysis of the transcriptome showed that the MM induced greater upregulation of contractile function, calcium handling, and mitochondria‐relevant genes in Corr compared to PA cells, such as ACTC1, ACTA1, MYL3, and COX6A1, which is in line with the promaturation effects (Figure 3E). In contrast, in the MM‐treated PA cells, the upregulated genes include: HSPA5, HSP90B1, BCL2, HSPA8, and CALR, which are known to regulate protein homeostasis and apoptosis in stressed cardiomyocytes (Figure 3F). Moreover, KEGG analysis identified enrichment of pathways, such as “Calcium signaling,” “Adrenergic signaling in cardiomyocytes,” “Protein processing in the endoplasmic reticulum,” and “Protein digestion and absorption” in both PA and Corr groups, however, the extent of enrichment differed between them (Figure S3C,D, Supporting Information). Reactome pathway enrichment analysis showed that MM treatment enhanced cardiomyocyte contractility in the Corr iPSC‐CMs, as indicated by the enrichment of pathways such as “Muscle contraction” and “Cardiac conduction.” In contrast, PA cells under MM treatment exhibited enrichment in ER stress‐related pathways, including “Unfolded Protein Response (UPR)” and “IRE1α activates chaperones” which indicates that MM promoted the maturity in Corr cells but has interrupted protein homeostasis and induced endoplasmic reticulum stress in the PA iPSC‐CMs (Figure S3E,F, Supporting Information).

**Figure 4:**
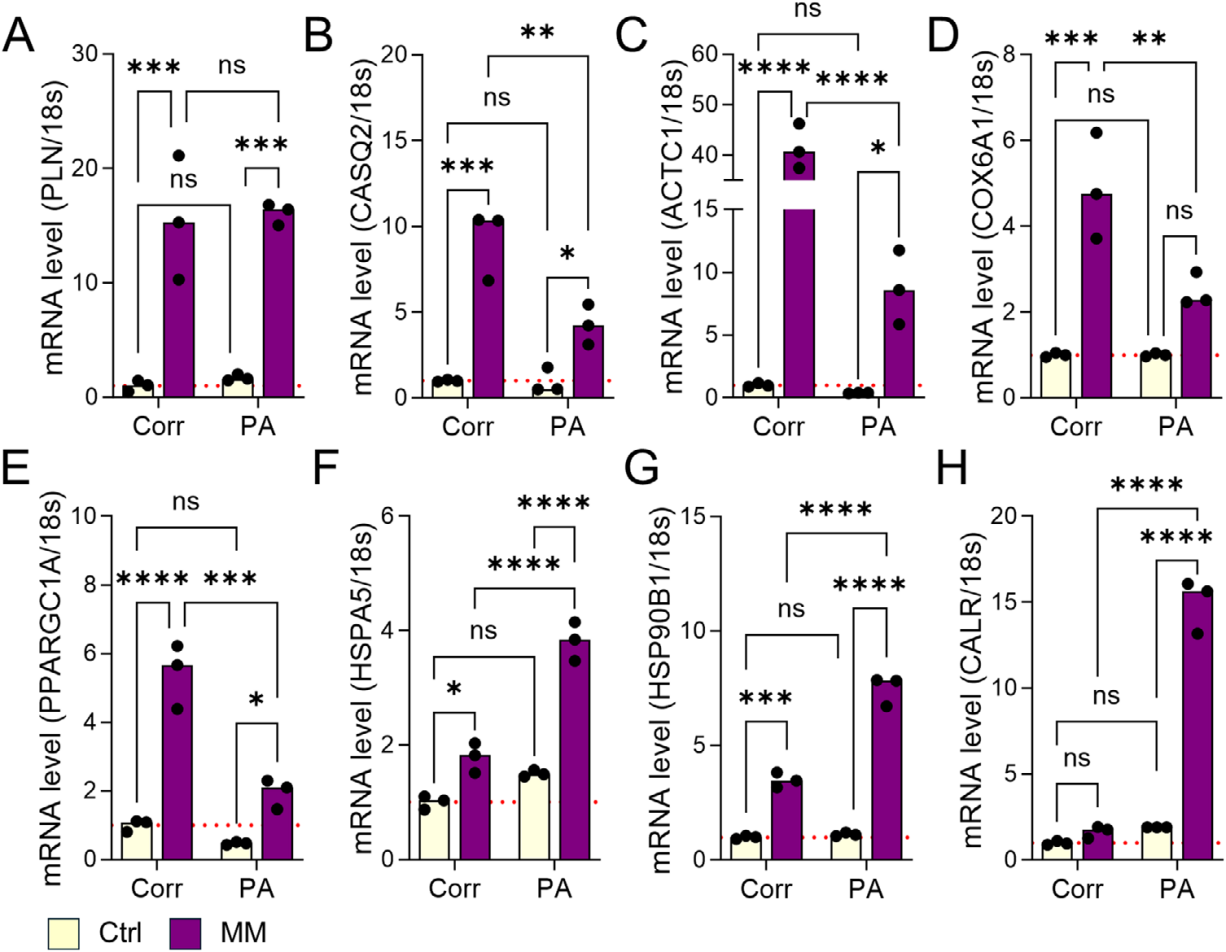
Validation of functional challenge‐induced DEGs in Corr and PA iPSC‐CMs with qPCR. A,B) Increased expression of calcium handling relevant genes PLN A) and CASQ2 B) in both Corr and PA iPSC‐CMs after MM treatment. C–E) MM treatment leads to a more significant increase of sarcomere and mitochondrial gene expressions, such as ACTC1 C), COX6A1 D), and PPARGC1A E), in Corr iPSC‐CMs compared to the PA group. F–H) Expression of protein homeostasis relevant genes, such as HSPA5 F), HSP90B1 G), and CALR H), were significantly upregulated in PA iPSC‐CMs after MM treatment, while much milder increases are observed in the Corr group. Results from 3 independent experiments. NS, no significance, *p < 0.05, **p < 0.01, ***p < 0.001, and ****p < 0.0001 by 2‐way ANOVA test followed by Holm–Sidak method.

Next, we validated the DEGs from RNA‐seq data by quantitative polymerase chain reaction (qPCR) (**Figure** **4**; and Figure S4, Supporting Information). Our results showed that many contractile functions and calcium handling relevant genes were up‐regulated in both MM‐treated Corr and PA cells, such as PLN and CASQ2 (Figure 4A,B). However, sarcomere and mitochondria genes, such as ACTC1, COX6A1, and PPARGC1A, showed a more pronounced increase in Corr CMs after MM treatment compared to PA group (Figure 4C–E). Instead, other proteostasis‐related genes, such as HSPA5, HSP90B1, and CALR, were more strongly upregulated in MM‐treated PA cells (Figure 4F–H). Notably, MM treatment significantly reduced MYH6 and increased MYH7 expression in Corr cells, but not in PA cells (Figure S4A,B, Supporting Information), indicating a pro‐maturation effect, marked by a MYH6‐to‐MYH7 switch, specifically in the Corr group CMs. Taken together, the mRNA expression profiling suggested MM treatment promoted functional maturation in Corr cells and enhanced proteostasis stress in PA cells.

### 2.4 Disrupted Proteostasis and Autophagic Flux in PLN R9C iPSC‐CMS under Maturation Stress

**Figure 5:**
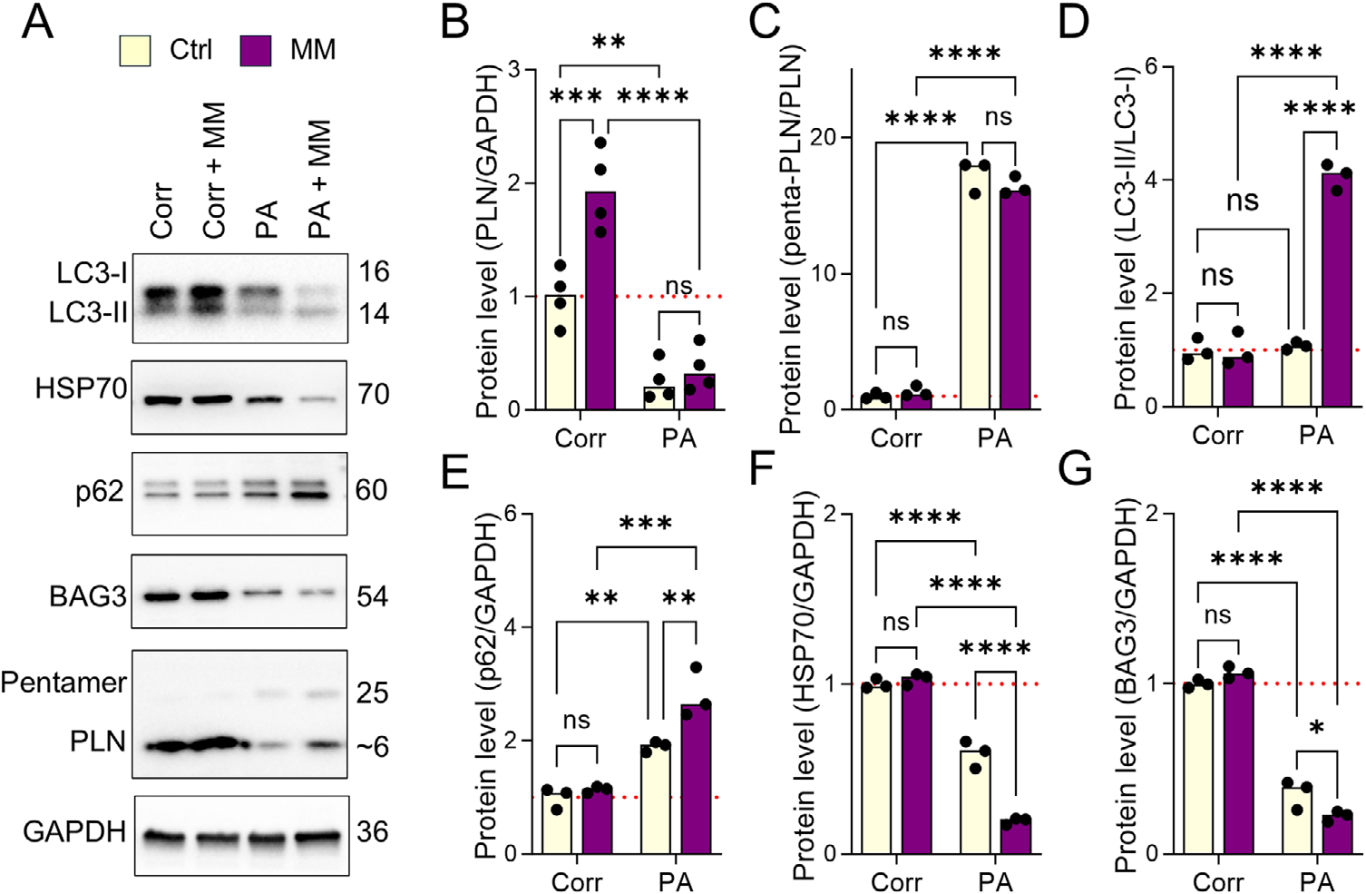
Functional challenge promotes polymerization of PLN in patient iPSC‐CMs. A) Western blot quantification of the abundance of monomer and pentamer PLN, autophagy markers, and key protein homeostasis markers in Corr and PA iPSC‐CMs before and after functional challenges. B) Heterozygous PLN R9C induced significant down‐regulation of PLN in iPSC‐CMs, and MM treatment enhanced the PLN level in Corr but not PA iPSC‐CMs. C) The PLN aggregation, as evidenced by the PLN pentamer/monomer ratio, was unchanged in MM‐treated Corr cells, yet was remarkably increased in the PA group, both before and after MM treatment. D,E) Autophagic flux, evidenced by the LC3‐II/I ratio and p62 expression, is unchanged in Corr cells before and after the functional stress by MM, while both were significantly increased in the PA iPSC‐CMs after MM treatment. F,G) PA iPSC‐CMs showed lower HSP70 and BAG3 protein levels compared to Corr cells, which were further down‐regulated in PA cells with MM treatment. Results from at least 3 independent experiments for all experiments. NS, no significance, *p < 0.05, **p < 0.01, ***p < 0.001, and ****p < 0.0001 by 2‐way ANOVA test followed by Holm–Sidak method.

To further validate the remodeling of autophagic signaling in PLN R9C cells, we used Western Blot to quantify the abundance of key proteins in Corr and PA iPSC‐CMs before and after MM treatment. Total PLN protein levels were markedly lower in PA cells than in Corr cells at baseline, despite unchanged RNA levels, possibly due to nonsense‐mediated RNA decay (NMD) activation and reduced mutant PLN stability (**Figures** 1I and **5**A).^[^ ^26^, ^27^ ^]^ Following MM treatment, PLN expression increased in Corr cells but remained unchanged in PA cells, reflecting enhanced demand for function regulation in more matured cardiomyocytes (Figure 5B). Notably, the PLN pentamer‐to‐monomer ratio was significantly elevated in PA cells both before and after MM treatment, suggesting enhanced pentamer formation, likely driven by reduced PLN R9C phosphorylation, impaired PLN‐SERCA2a interaction, and interrupted PLN degradation (Figures 5C; and S4C, Supporting Information). Given autophagy is essential for the lysosomal degradation of PLN, we assessed autophagic flux using the LC3‐II/LC3‐I ratio. At baseline, PA and Corr cells showed similar autophagic activity. However, MM treatment markedly increased the LC3‐II/LC3‐I ratio in PA cells, indicating heightened autophagic stress, while Corr cells showed no significant change (Figure 5D). Similarly, the autophagic stress marker p62 accumulated specifically in MM‐treated PA cells, pointing to a blockage of autophagic flux at the degradation stage (Figure 5E). Consistent with this, levels of HSP70 and BAG3, key regulators of protein homeostasis, were significantly reduced in PA cells compared to the Corr group, underscoring disrupted proteostasis in PLN R9C iPSC‐CMs (Figure 5F–G). We also evaluated autophagic flux using additional markers (Figure S4D–G, Supporting Information), and the levels of LC3‐II and ATG5 indicated that MM treatment led to an increase of autophagosome accumulation and autophagic flux in Corr but not PA cells, while the Ser.p758‐ULK1/ULK1 level showed increased phosphorylation of ULK1 by mTOR specifically in MM treated PA cells, which disrupts the interaction between ULK1 and AMPK and inhibits autophagy.^[^ ^28^, ^29^ ^]^

### 2.5 Activation of Autophagy Restores Proteostasis and Cardiac Function in PA iPSC‐CMs

**Figure 6:**
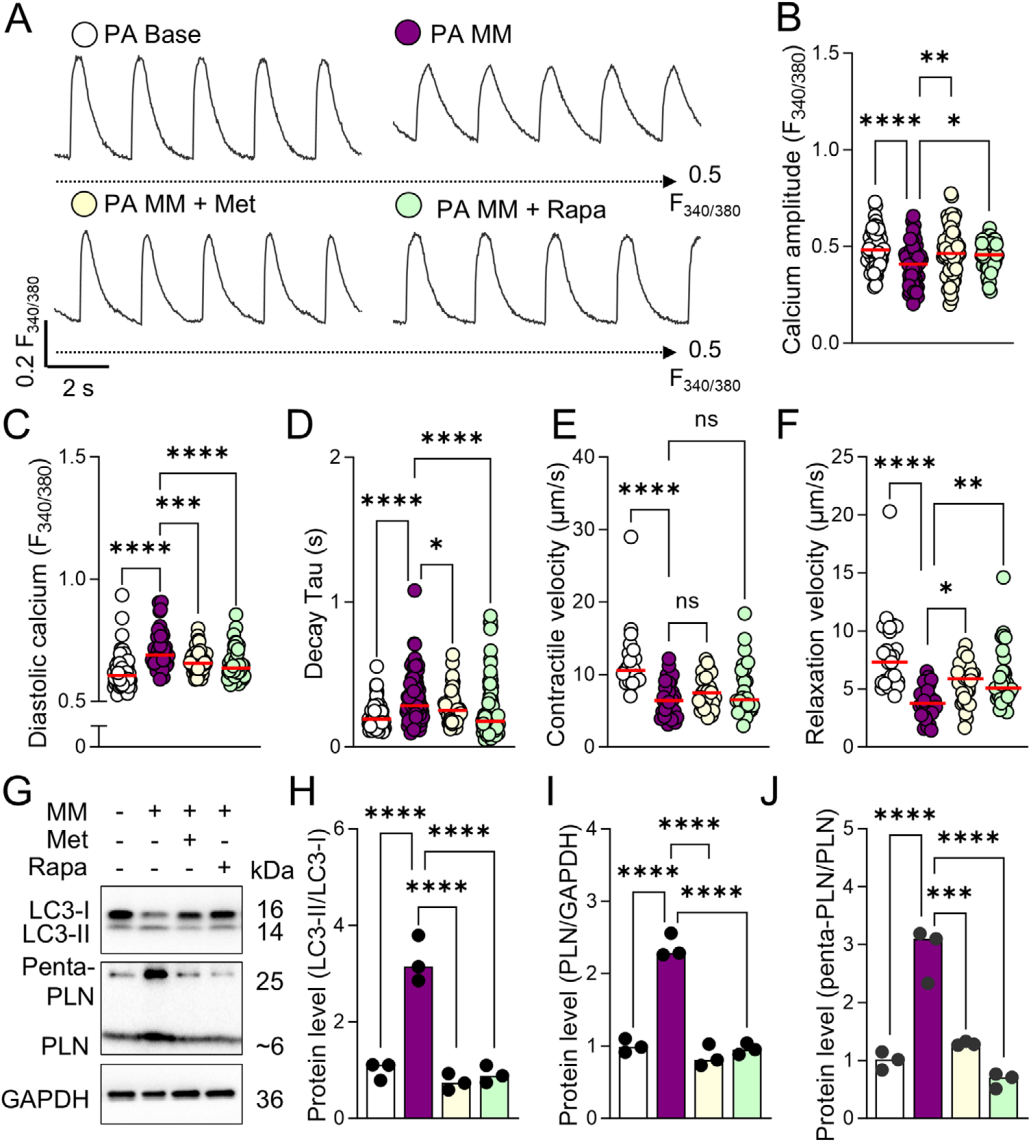
Activation of autophagy restored cardiac function of patient‐specific PLN R9C iPSC‐CMs. A) Representative Fura‐2 AM calcium trace in PA iPSC‐CMs at baseline, after MM treatment, and MM treatment in the presence of 2 mm metformin (Met) or 0.5 µm rapamycin (Rapa). The black dashed line indicates the F340/F380 ratio of 0.5. B–D) Quantification of calcium transient amplitude B), diastolic calcium level C), and calcium recycling rate D). *N* = 64, 56, 68, and 64 cells of PA, PA MM, PA MM + Met, and PA MM + Rapa iPSC‐CMs from at least 3 independent experiments. E,F) TFM measurement of contraction D) and relaxation E) velocities. *N* = 26, 31, 32, and 29 ROIs of PA, PA MM, PA MM + Met, and PA MM + Rapa iPSC‐CMs from at least 3 independent experiments. NS, no significance, *p < 0.05, **p < 0.01, and ****p < 0.0001 by 1‐way ANOVA test followed by Holm–Sidak method. G) Western blot quantification of the abundance of LC3‐II, LC3‐I, as well as monomer and pentamer PLN in PA, PA MM, PA MM + Met, and PA MM + Rapa groups. H–J) Activation of autophagy by Met and Rapa mitigated the functional stress‐induced autophagic stress, indicated by the LC3‐II/LC3‐I ratio H) and the aggregation of PLN (I) and penta‐PLN (penta‐PLN/mono‐PLN, J) in patient iPSC‐CMs. Results from 3 independent experiments. ****p < 0.001 and ****p < 0.0001 by 1‐way ANOVA test followed by Holm–Sidak method.

RNA‐seq and functional analyses identified disrupted protein homeostasis as a central mechanism driving stress‐induced pathogenesis in PLN R9C cardiomyocytes. To test whether enhancing autophagic signaling could mitigate these deficits, we treated PA iPSC‐CMs with metformin (Met, 2 mm) or rapamycin (Rapa, 0.5 µm), known autophagy inducers via AMPK activation and mTOR inhibition, respectively, during a 7‐day promaturation medium (MM) challenge.^[^ ^30^, ^31^ ^]^ Both treatments partially restored functional performance in PA iPSC‐CMs, evidenced by increased calcium transient amplitude, accelerated calcium recycling, normalized diastolic calcium levels, and enhanced contractile and relaxation velocities (**Figure** **6**A–F). Sarcomere structure analysis further revealed improved regularity and arrangement following Met and Rapa treatment in MM‐treated PA iPSC‐CMs (Figure S5A–C, Supporting Information). We also assessed the beneficial effect of metformin and rapamycin on the mitochondrial structure with transmission electron microscopy (TEM), indicating significantly reduced mitochondrial damage in stressed PLN R9C cells (Figure S5D–E, Supporting Information). To elucidate the mechanism, western blot analysis confirmed autophagy activation, with a significant reduction in the LC3‐II/LC3‐I ratio in Met‐ and Rapa‐treated PA cells, indicating restored autophagic flux (Figure 6G,H). Consequently, PLN protein levels as monomer and pentamer formats both decreased in treated cells compared to the MM‐only group (Figure 6F,H). Collectively, these findings demonstrate that activating autophagy partially rescues morphological and functional deficits in PLN R9C iPSC‐CMs, likely by restoring degradation of mutant PLN and mitigating stress‐induced remodeling.

### 2.6 PLN R9C Knock in iPSC‐CMs Recapitulated the Functional Deficits in Patient‐Derived Cells

**Figure 7:**
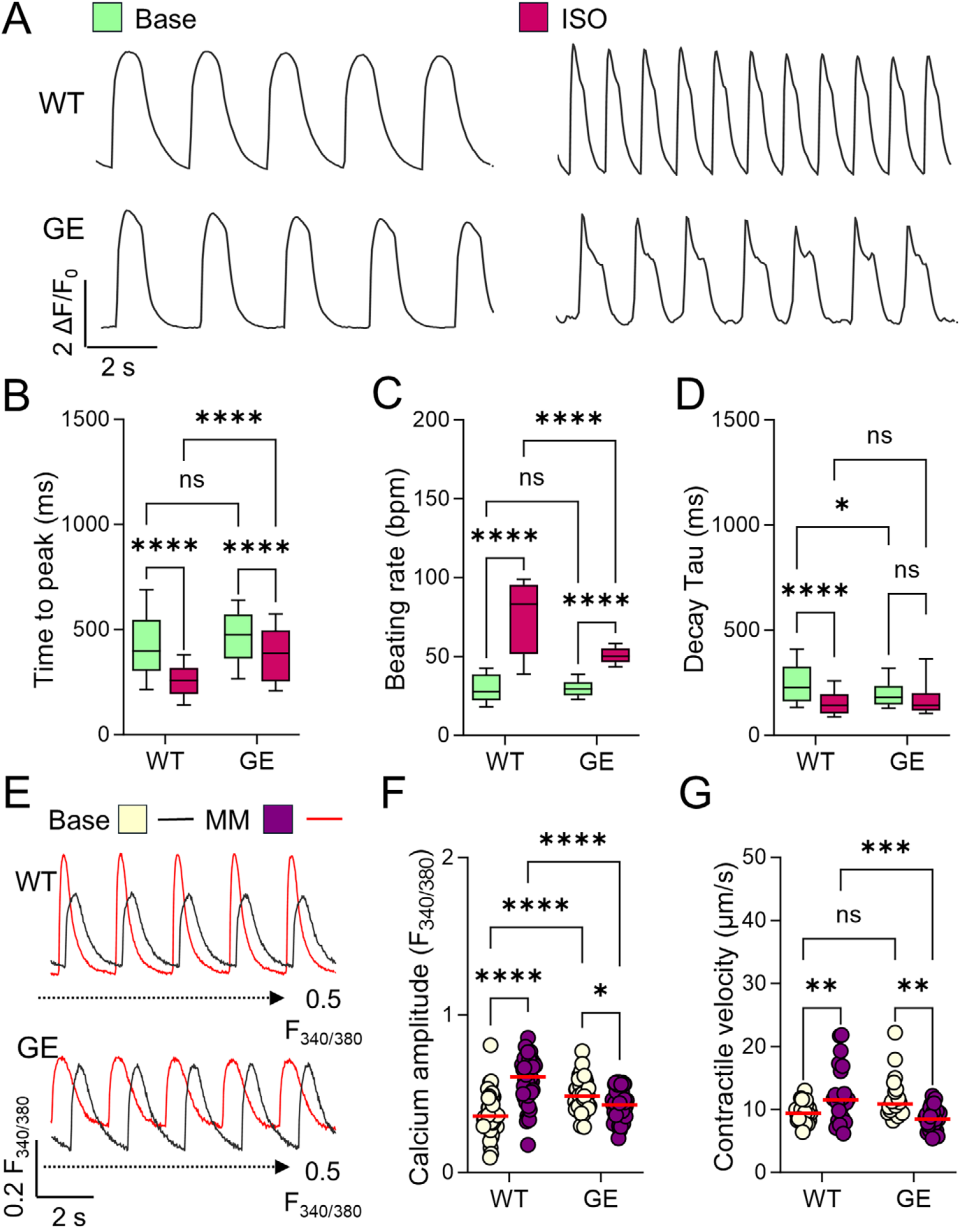
The functional profiling of PLN R9C mutation knock‐in iPSC‐CMs. A) Representative Fluo‐4 calcium transient trace in wild type (WT) and PLN R9C knock‐in (GE) iPSC‐CMs before and after ISO treatment. B–D) β‐adrenergic activation induced no or milder changes in calcium handling capacity, such as transient rise time B), transient decay Tau C), and beating rate D) in GE iPSC‐CMs compared to WT cells. *N* = 132, 105, 143, and 137 cells of WT, GE, WT ISO, and GE ISO groups from at least 3 independent experiments. E) Representative Fura‐2 AM of paced calcium transient trace in WT and GE iPSC‐CMs before (black line) and after MM treatment (red line). The black dashed line indicates the F340/F380 ratio at 0.5. F) Comparison of calcium transient amplitude with Fura‐2 imaging. *N* = 51, 46, 51, and 52 cells of WT, GE, WT MM, and GE MM groups from at least 3 independent experiments. G) Traction force microscopy analysis showed slightly increased contractility in GE iPSC‐CMs at the basal level compared to WT cells, while MM treatment enhanced the contractility of WT but not GE iPSC‐CMs. *N* = 27, 24, 26, and 26 ROIs of WT, GE, WT MM, and GE MM groups from at least 3 independent experiments. NS, no significance, *p < 0.05, **p < 0.01, ***p < 0.001, and ****p < 0.0001 by 2‐way ANOVA test followed by Holm–Sidak method.

To validate our findings on the pathological mechanism of PLN R9C, we used CRSIPR to introduce the heterozygous PLN R9C knock‐in (GE, genome‐edited cells carrying R9C mutation) into two wild type iPSCs (WT, iPSCs from healthy donors). Both WT and their isogenic GE iPSCs showed comparable differentiation efficiency into iPSC‐CMs (Figure S6A,B, Supporting Information). Consistent with our observations in PA iPSC‐CMs, GE iPSC‐CMs exhibited near‐normal morphology and function at baseline. However, spontaneous calcium transient recording revealed impaired β‐adrenergic signaling in GE cells compared to WT cells. Specifically, treatment with ISO elicited only a modest increase in transient rise time and beating rate, with no change in calcium recycling rate, indicating a blunted response (**Figure** **7**A–D). This aligned with reduced PLN phosphorylation in GE cells relative to WT cells (Figure S6C–E, Supporting Information).

**Figure 8:**
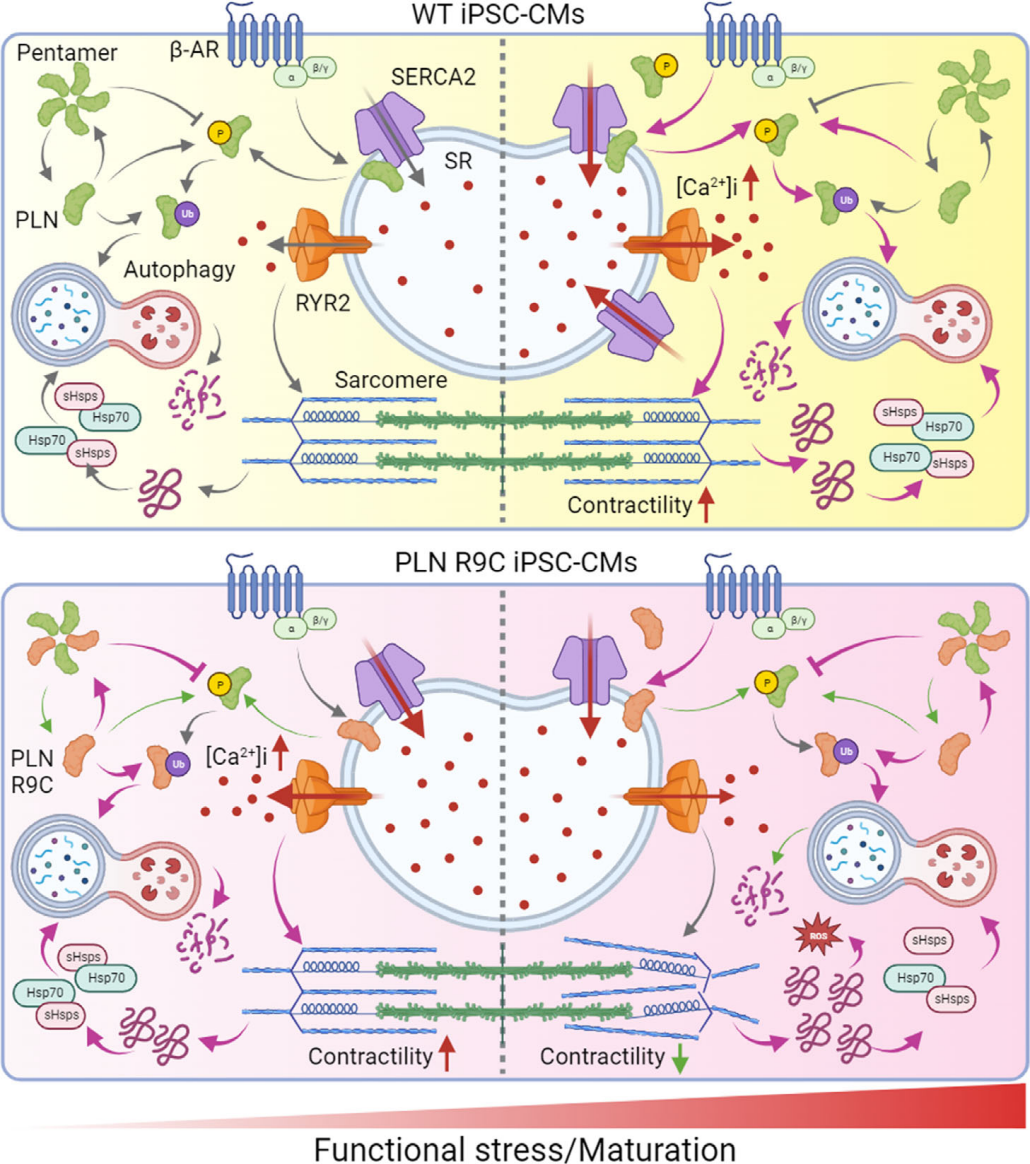
A schematic diagram of the pathological mechanism of PLN R9C in DCM. At baseline, PLN R9C mutation exhibits lower binding affinity to SERCA, increasing SERCA availability and SR calcium load, which elevates calcium transients and contractility. Moreover, mutant PLN preferentially forms pentamers, hindering phosphorylation even under ISO stimulation, leading to minimal p‐PLN detection and impaired β‐adrenergic response. Under cardiac stresses, demands on protein quality control intensify. The overwhelming autophagic pathway in PLN R9C CMs fails to fully resolve the accumulation of misfolded protein and PLN pentamers, resulting in elevated ER stress, sarcomere disarray, and functional remodeling. Activation of autophagic flux can partially restore these deficits, indicating a key pathological mechanism and therapeutic target in PLN R9C cardiomyopathy.

To further probe stress‐induced effects, we subjected WT and GE iPSC‐CMs to MM treatment for 7 days (Figure 7E; and Figure S6F, Supporting Information). Similar to PA cells, MM‐treated GE cells exhibited compromised sarcomere integrity alongside functional deficits, including reduced calcium transient amplitude, elevated diastolic calcium, and decreased contractile and relaxation velocities (Figure 7F,G; and Figures S6F–H,S7L,M, Supporting Information). RNA‐seq profiling and pathway enrichment analysis further revealed disrupted autophagic flux in MM‐treated GE cells, mirroring findings in PA cells (Figure S7A–D, Supporting Information). Western blot analysis confirmed the finding with enhanced PLN pentamer formation and disrupted autophagic flux under functional stress (Figure S7E–K, Supporting Information). Collectively, these findings indicate that cardiomyocytes harboring the PLN R9C mutation exhibit reduced adaptability to adrenergic signaling challenges and heightened proteostasis stress under increased functional demand. This impaired response drives pathological structural and functional remodeling over time (**Figure** **8**).

## 3 Discussion

So far, multiple PLN mutations and genetic variants have been identified in patients with cardiomyopathy and heart failure. These include: 1) missense mutations such as p.Arg9Cys (R9C), p.Arg9His (R9H), and p.Arg25Cys (R25C); 2) nonsense mutations such as p.Leu39Ter (L39X) and p.Glu2Ter (E2X); and 3) the deletion mutation p.Arg14del (R14del), alongside other variants.^[^ ^14^ ^]^ Among these, PLN R14del is the most extensively studied, characterized in various animal and cell line models. Current evidence indicates that PLN R14del enhances association with SERCA2a, leading to increased SERCA2a inhibition, reduced sarcoplasmic reticulum (SR) calcium uptake, and diminished contractility. The absence of Ser‐16 phosphorylation in PLN R14del renders this inhibition chronic, which cannot be relieved by β‐adrenergic stimulation.^[^ ^32^ ^]^ This slowed SR calcium uptake elevates diastolic calcium levels, promoting arrhythmogenesis in affected patients.^[^ ^12^, ^33^, ^34^ ^]^ Similarly, a previous study showed PLN R25C mutation exhibits enhanced SERCA2a binding, resulting in super‐inhibition, decreased SR calcium load, reduced calcium transients, and impaired contractility. Unlike R14del, PLN R25C retains PKA‐mediated phosphorylation, allowing β‐adrenergic stimulation to partially alleviate SERCA2a inhibition.^[^ ^7^ ^]^ However, excessive β‐adrenergic activation increases CaMKII activity and hyper‐phosphorylates RyR2 at Ser‐2814, promoting arrhythmia risk under stress. As the focus of this study, PLN R9C is associated with early‐onset severe DCM without an arrhythmic phenotype.^[^ ^9^ ^]^ Unlike R14del and R25C, which amplify SERCA2a inhibition, PLN R9C nearly abolishes its binding to and inhibition of SERCA2a, highlighting its distinct pathogenic mechanism. Previous studies showed that over‐expression of mutant PLN R9C in both animal and cell models resulted in decreased SERC2a inhibition, acute inotropic and lusitropic effects, and blunted β‐adrenergic responsiveness, which likely contributes to long‐term calcium dysregulation and heart failure.^[^ ^16^, ^18^ ^]^ These studies suggest a dominant negative effect of PLN R9C during pathogenesis. Nevertheless, the intricate molecular pathways driving PLN R9C pathogenesis remain incompletely understood in human cardiomyocytes.

In our current study, we generated R9C DCM iPSC lines and isogenic controls to study the PLN R9C mutation in human cardiomyocytes. Our results partially align with prior iPSC studies, showing attenuated β‐adrenergic signaling in PLN R9C KI iPSC‐CMs.^[^ ^20^ ^]^ At baseline, DCM iPSC‐CMs displayed near‐normal beating rates and calcium amplitudes, with slightly faster calcium recycling (Decay Tau) and relaxation velocity, consistent with reduced PLN‐SERCA2a interaction enhancing SERCA activity. Indeed, Fura‐2 imaging confirmed lower diastolic calcium in PA cells, supporting enhanced calcium reuptake (Figure S2C, Supporting Information). Moreover, in our observation, key functional deficits of PA iPSC‐CMs displayed a blunted β‐adrenergic response, with ISO eliciting only modest improvements in calcium handling and contractility compared to controls. Indeed, previous studies suggest that the PLN‐R9C conformational change impairs PKA binding and phosphorylation, reducing phosphorylated PLN by 60%.^[^ ^23^ ^]^ Additionally, the PLN‐R9C‐PKA complex dissociates more slowly than the wild‐type complex, potentially trapping activated PKA and further dampening β‐adrenergic signaling. This was corroborated by reduced p.Ser16‐PLN levels in DCM iPSC‐CMs post‐ISO treatment compared to controls. Although DCM iPSC‐CMs showed slightly enhanced baseline performance, their response to β‐adrenergic stimulation was significantly diminished relative to controls, suggesting a mismatch between contractility and functional demand in DCM hearts under stress.

To evaluate the response of Corr and PA iPSC‐CMs to functional stress, we applied maturation medium (MM) to promote functional maturation.^[^ ^24^, ^35^ ^]^ In Corr cells, MM upregulated genes linked to contractility and calcium handling (e.g., ACTC1, CASQ2), as shown by RNA‐seq, and enhanced contractility and calcium dynamics. In contrast, MM induces protein homeostasis stress in patient‐derived iPSC‐CMs, worsening disease phenotypes.^[^ ^25^ ^]^ Indeed, RNA‐seq and GO‐term analysis revealed interrupted autophagic and proteostasis pathways (e.g., protein degradation and absorption) in MM‐treated PA cells, but not Corr cells, which is consistent with their elevated baseline contractility and reduced adaptability to increased functional demand (due to diminished β‐adrenergic responsiveness). Previous studies have shown that PLN R9C mutation affects the dynamics of PLN‐SERCA2a complex and PLN phosphorylation, which affects PLN polymerization and ubiquitination.^[^ ^26^ ^]^ Our results show significantly lower PLN monomer levels in patient cells than their isogenic controls, indicating mutant PLN instability due to protein quality control mechanisms. However, the PLN pentamer levels are significantly higher in mutant cells, suggesting PLN R9C induced a change of the monomer‐pentamer balance, likely through a conformational change that reduces SERCA2a binding and leads to accumulation of “free” PLN, thereby promoting pentamer formation.^[^ ^4^ ^]^ Post‐MM treatment, both monomer and pentamer PLN levels increased markedly in mutant cells, likely due to 1) PLN R9C mutation is known to alter its cytoplasmic domain and disrupt the interaction between PLN and SERCA2a, which increases the free monomeric PLN and facilitates pentamer formation.^[^ ^19^ ^]^ 2) Fura‐AM calcium measurements showed elevated diastolic calcium levels in stressed PLN‐R9C cells (Figure S2C, Supporting Information), associated with increased CaMKII activation. This, in turn, may enhance Thr17 phosphorylation of PLN and promote pentamer formation.^[^ ^4^ ^]^ 3) Impaired proteostasis under functional stress fails to clean up the mutant PLN, leading to the accumulation of both PLN monomers and pentamers.^[^ ^19^ ^]^ Beyond PLN, deficiencies in the protein quality control system likely limited renewal of critical cardiac proteins, such as myofilament components, contributing to sarcomere disarrangement in mutant cells under prolonged stress.

Taken together, these findings indicate that the PLN R9C mutation may drive early‐onset DCM through two mechanisms. First, it disrupts PKA binding and phosphorylation, desensitizing mutant cells to β‐adrenergic signaling. Although these cells function almost normally at baseline, their response to β‐adrenergic stimulation fails to meet increased demands under stress. To offset this deficiency, the body boosts adrenaline signaling, briefly improving cardiac output, but chronic overactivation drives long‐term heart remodeling, including cardiomyocyte loss, fibrosis, and eventual heart failure.^[^ ^36^, ^37^ ^]^ Second, the mutation nearly abolished PLN's inhibition of SERCA2a, increasing baseline cardiac contractility. This sustained increase of cardiac function at the basal level strains the protein quality control system. Under further functional stress (e.g., MM treatment or increased cardiac output), mutant cells are subjected to increased challenges maintaining proteostasis and autophagic flux, leading to accumulation of misfolded protein, ER stress, ROS production, and mitochondrial dysfunction, which are key pathological mechanisms underlying the detrimental effects of the PLN‐R9C mutation (Figure 8).

Autophagy is a highly conserved cellular process that maintains homeostasis by degrading long‐lived or damaged proteins and organelles within lysosomes.^[^ ^38^ ^]^ In cardiomyocytes, basal autophagic activity is low but can increase as a protective response to cardiac stress, such as energy restriction or heightened functional demand, which counteracts toxic protein aggregation.^[^ ^39^ ^]^ Deficiencies in autophagy, often arising from mutant or misfolded proteins, are implicated in various cardiac diseases, including dilated cardiomyopathy (DCM), hypertrophic cardiomyopathy (HCM), diabetic cardiomyopathy, desmin‐related cardiomyopathy, and Danon disease.^[^ ^40^ ^]^ Given its pivotal role, therapeutic strategies enhancing autophagy, such as overexpression of proteins (like ATG7 or UBC9) and activation with drugs (rapamycin, metformin, or resveratrol) have been explored.^[^ ^41^, ^42^, ^43^, ^44^, ^45^ ^]^ And studies have demonstrated that autophagy activation reduces protein aggregation, hypertrophy, and fibrosis, while improving cardiac function and lifespan in animal models of DCM, HCM, and age‐related cardiomyopathy.^[^ ^46^, ^47^, ^48^ ^]^

In our study, iPSC‐CMs harboring PLN R9C mutation exhibited near‐normal basal function despite markedly reduced PLN protein levels, suggesting that mutant PLN was degraded by the protein quality control system to sustain cardiomyocyte homeostasis. However, prolonged functional stress overwhelmed this compensation, as stressed mutant cells showed blocked autophagic flux (increased LC3‐II/LC3‐I and p62), significant PLN accumulation in pentamer format, alongside functional deficits. Recent studies of other PLN mutations similarly report aberrant unfolded protein responses and myofilament changes, aligning with our observations.^[^ ^49^, ^50^ ^]^ Moreover, PLN degradation in cardiomyocytes relies on phosphorylation and autophagic pathways, reinforcing a shared mechanism of signaling remodeling in PLN mutation‐driven DCM.^[^ ^26^, ^51^ ^]^

To mitigate PLN R9C‐induced functional deficits in patient‐specific iPSC‐CMs, we evaluated rapamycin, an FDA‐approved mTOR inhibitor, and metformin, an AMPK activator.^[^ ^52^, ^53^ ^]^ We found that activating autophagic signaling reduced mutant PLN accumulation in diseased cells. Notably, our study is the first to demonstrate that metformin and rapamycin treatments partially restore sarcomere organization and cardiac function in functionally stressed DCM patient‐specific iPSC‐CMs carrying PLN R9C. However, β‐adrenergic signaling responsiveness remained impaired, likely due to persistently lower PLN levels in treated DCM cells compared to controls.

Although this study takes advantage of patient‐specific iPSC‐CMs and their isogenic control lines to identify protein homeostasis deficiency as a key contributor to DCM pathogenesis in PLN R9C cells and to suggest potential therapeutic targets, it remains limited by the exclusive use of an in vitro modeling system and the availability of only one patient‐derived line. Future studies could further advance this field in several important directions: 1) The application of innovative 3D culture models, such as engineered heart tissue (EHT), would enable the investigation of long‐term structural and functional remodeling of cardiomyocytes, as well as intercellular signaling and interactions, under more physiological conditions. This approach would also provide a broader spectrum of readouts, including metabolic alterations, apoptosis, fibrosis, and arrhythmogenesis.^[^ ^20^ ^]^ 2) Accumulating evidence has highlighted impaired autophagy as a central mechanism in PLN mutation‐related cardiomyopathies.^[^ ^51^, ^54^ ^]^ Therefore, validating the therapeutic benefits of autophagy pathway activation in PLN R9C transgenic mice would provide compelling in vivo evidence supporting its pathogenic role and translational potential.^[^ ^9^, ^16^ ^]^ Moreover, we observed reduced expression of PLN and several key sarcomere proteins in PLN R9C iPSC‐CMs compared with WT cells, which may underlie the functional deficiency of mutant cells. Further investigation using PLN overexpression iPSC models may clarify how mutant PLN exerts dominant‐negative effects on PLN degradation and whether this disrupts the translation or stability of proteins essential for contractile homeostasis, offering new insight into PLN R9C‐associated DCM.

In summary, our findings establish overwhelmed autophagic flux as a central mechanism driving PLN R9C‐associated cardiomyopathy under functional stress. We show that enhancing autophagic signaling restores autophagic flux, decreases PLN accumulation, prevents structural remodeling, and improves calcium handling and contractility in patient‐derived cells under stress. These results suggest a promising therapeutic strategy for DCM patients with PLN mutations.

## 4 Experimental Section

### Recruitment of the Dilated Cardiomyopathy Patient with PLN R9C Mutation

A male patient (age = 44) carrying a PLN R9C mutation, diagnosed with severe dilated cardiomyopathy and markedly depressed cardiac function (EF ≈25%–35%) was recruited and consented to the current study. The recruitment of patient tissue samples followed the IRB#29 904 of Stanford University, approved by the SU ethics committee. The skin biopsy was isolated and rinsed in phosphate‐buffered saline (PBS). The skin biopsy was then minced and digested in DMEM medium (Dulbecco's modified Eagle medium) containing 1 mg mL^−1^ collagenase I at 37 °C for 6 h. Dermal fibroblasts were collected by centrifugation at 300 × g for 5 min, and then maintained with DMEM containing 10% fetal bovine serum (FBS), 100 U mL^−1^ Penicillin, and 100 U mL^−1^ Streptomycin. The fibroblasts were used for reprogramming within 5 passages.

### Reprogramming of Patient‐Specific iPSCs

Patient‐specific iPSCs were generated from fibroblasts using CytoTune‐iPS 2.1 Sendai Reprogramming Kit (Thermo Fisher) following the manufacturer's instructions. 2 days before virus transduction, dermal fibroblasts were maintained in DMEM + 10% FBS and reseeded on a 6‐well plate at a density of ≈1–2 ×10^5^ cells per well. When cells were ≈30%–60% confluent, Sendai Virus mixture (expressing KOS, hL‐Myc, hKlf4) was applied to the cell culture with MOIs of 5 for KOS, 5 for hL‐Myc, and 3 for hKlf4. The culture medium was replaced with xeno‐free fibroblast medium 24 h after transduction. The cells were in the same well before passage. After 7 days of transduction, fibroblast cells were harvested and seeded on Matrigel‐coated 6‐well plates. After 24 h, the culture medium was changed to Essential 8 (E8) (Giboco), which was replaced every day until single iPSC clones were observed in the plate. At least 6 clones were picked and kept in culture for at least 10 generations. The quality of these iPSCs was validated by immuno‐staining of pluripotent markers, such as SSEA‐4, Nanog, SOX2, and OCT4, as well as the in vitro 3‐germ layer differentiation assays with STEMdiff Trilineage Differentiation Kit (StemCell). The successful differentiation of ectoderm, endoderm, and mesoderm was confirmed by immunostaining of γ‐tubulin (TUBB3), SOX17, and brachyury. The current study used 2 different patient iPSC clones from this DCM patient and their respective isogenic corrected lines for downstream analysis.

### Generating Isogenic Corrected iPSC Lines with Genome Editing by CRISPR/Cas9

CRISPR/Cas9‐mediated gene editing was performed to correct the PLN R9C mutation. The PLN‐targeting sgRNA and single‐stranded oligodeoxynucleotide (ssODN) repair template were designed using Benchling (https://benchling.com/) (Table S1, Supporting Information). RNP complexes were prepared by gently mixing Opti‐MEM (Thermo Fisher), 20 pmol EnGen Spy Cas9 NLS (New England Biolabs), and 100 pmol TrueGuide Synthetic gRNA (Thermo Fisher), followed by incubation at room temperature for 20 min. After incubation, 40 pmol ssODN (IDT) was added to the mixture. During the incubation, cells were washed once with DPBS and treated with 1 mL Gentle Cell Dissociation Reagent (STEMCELL Technologies). After cell dissociation, the dissociation reagent was removed, and cells were resuspended in E8 medium supplemented with 10 µm Y‐27632. The cells were centrifuged at 150 × g for 3 min, resuspended, and counted. The Neon Transfection System (Thermo Fisher) was used for electroporation according to the manufacturer's instructions. For each reaction, 1 × 10⁶ cells were resuspended in 100 µL Buffer R and gently mixed with the prepared RNP mixture. A Neon tube containing 3 mL Buffer E2 was set up for use with a 100 µL Neon tip. The 100 µL RNP per cell mixture was transferred to the tip, and electroporation was performed using the following parameters: 1650 V, 10 ms, 3 pulses. Immediately after electroporation, cells were transferred into a Matrigel‐coated plate containing pre‐warmed E8 medium with 10 µm Y‐27632 and incubated at 37 °C with 5% CO_2_/5% O_2_ in a humidified incubator. The medium was changed daily until colonies were ready for clone picking and genotyping. Surviving single colonies were manually picked after 2–3 weeks, expanded in 24‐well plates, and screened for successful genome editing by PCR genotyping. Similarly, the PLN R9C mutation was introduced into 2 different WT iPSC lines for the generation of genome‐edited (GE) isogenic controls in this study.^[^ ^22^ ^]^

### Maintenance and Differentiation of Human iPSCs

The human iPSC lines were maintained daily with E8 on Matrigel‐coated plates at 37 °C with 5% oxygen and 5% CO_2_ without antibiotics. Cells were dissociated using DPBS with 0.5 mm EDTA and reseeded in E8 with 10 µm Y‐27632 dihydrochloride. After 24 h, fresh E8 medium without Y‐27632 was changed every 24–48 h until passage. For cardiomyocyte differentiation, iPSCs were cultured to confluency and differentiated into beating cardiomyocytes using a monolayer differentiation protocol as previously described. Briefly, confluent iPSCs were rinsed with DPBS and treated with 6 µm CHIR99021 in RPMI 1640 and B27 supplement minus insulin (RPMI+B27‐Insulin) for 48 h. Subsequently, cells were switched back to RPMI+B27‐Insulin for 24 h and then exposed to 5 µm IWR with RPMI+B27‐Insulin for 48 h. Afterward, cells were placed in RPMI+B27‐Insulin for 48 h and finally cultured in RPMI 1640 and B27 supplement for 48 h. The medium was changed every 48 h until beating cells were observed.

### In Vitro Functional Challenge and Drug Treatment of iPSC‐CMs

The maturation medium (MM) was prepared as previously described.^[^ ^24^ ^]^ RPMI‐glucose medium was supplemented with 3 mm D‐(+)‐glucose (Sigma), 10 mm sodium L‐lactate (Sigma), 5 mm creatine monohydrate (Sigma), 2 mm taurine (Sigma), 2 mm L‐carnitine hydrochloride (Sigma), 500 mm ascorbic acid 2‐phosphate sesquimagnesium salt hydrate (Sigma), 1× MEM nonessential amino acid solution (Gibco, New York), 5 mg mL^−1^ vitamin B12 (Sigma), 0.82 mm biotin (Sigma), 0.5% AlbuMAX I lipid‐rich BSA (Gibco), and 1% KnockOut Serum replacement (Gibco), 1×B27 supplement, then pH adjusted to 7.4 and 0.2 µm‐filter sterilized (Millipore, Burlington, MA). In the current study, iPSC‐CMs were shifted to MM for 7 days prior to analysis. For the measurement of acute consequences of β‐adrenergic activation, isoproterenol (ISO) was added to the cells 5 min prior to imaging at a final concentration of 100 nm. Protein samples for phosphorylation measurement were collected 30 min after ISO treatment. For the rescusing of functional challenges by MM, cells were supplemented with 0.5 µm of rapamycin or 2 µm of metformin throughout the treatment of MM.

### RNA Extraction of mRNA Expression Quantification by qPCR

Total RNA was extracted from the iPSCs and iPSC‐derived cardiomyocytes (iPS‐CMs) using TRIzol according to the manufacturer's instructions. RNA was purified using Direct‐zol RNA MicroPrep columns and treated with DNase I. The quality of RNA samples was determined with a NanoDrop 8000 spectrophotometer. DNase‐treated RNA was reverse transcribed to cDNA for qPCR using iScript reverse transcription supermix according to the manufacturer's instructions. qPCR was performed using iTaq universal SYBR green supermix with 0.3 µm of each primer and analyzed in triplicate with the QuantStudio5 analyzer. Relative gene expression was calculated using the 2^‐ΔΔ^CT method. The qPCR primers used in the current study are listed in Table S1 (Supporting Information).

### Protein Extraction and Western Blot Analysis

Whole cell protein lysates were extracted from iPSCs and iPS‐CMs using Pierce RIPA buffer supplemented with complete ULTRA EDTA‐free protease inhibitor cocktail. Total protein concentrations were determined using the Pierce BCA Protein Assay on a Synergy HT BioTek microplate reader at 562 nm and Gen5 v2.04 software with the standard curve method. 10 µg of protein was mixed with 5× loading buffer and boiled for 10 min prior to loading on a 4%–15% Mini‐PROTEAN TGX stain‐free gel. Protein samples were fractionated by SDS–polyacrylamide gel electrophoresis and transferred to polyvinylidene fluoride (PVDF) membranes using a Trans‐blot Turbo (Bio‐Rad) at 1.3 A, 25 V. Membranes were placed in blocking solution (5% milk in TBS with 0.1% Tween 20, pH 7.6) for 60 min, then incubated with primary antibodies in blocking solution at 4 °C overnight. Following three TBST washes, membranes were incubated with secondary horse anti‐mouse IgG at 1:10,000 or goat anti‐rabbit at 1:2500 HRP‐linked antibody in blocking solution. After washing three times, bands were visualized using chemiluminescent Clarity Max Western ECL Substrate. The Western blot images were analyzed using Fiji ImageJ software. The antibodies used in the current study are listed in Table S2 (Supporting Information).

### RNA‐seq: Sample Preparation and Data Analysis

cDNA Libraries were prepared following the DNBSEQ Eukaryotic Stranded Transcriptome library preparation pipeline. mRNA was enriched with Oligo dT selection and fragmentation. First‐strand cDNA was synthesized using random N6‐primed reverse transcription and dUTP for second‐strand synthesis. Ends were repaired, 3′ adenylated, and adapters ligated. cDNA was amplified by PCR, denatured by heat, and cyclized by splint oligo and DNA ligase. The sequencing of the cDNA libraries was done on the DNBSEQ platform. Sequencing reads were inspected by FastQC (v0.12.1, http://www.bioinformatics.babraham.ac.uk/projects/fastqc/) and trimmed through Trim Galore (v0.6.7, https://www.bioinformatics.babraham.ac.uk/projects/trim_galore/) to retain high‐quality clean reads for subsequent analysis. All clean reads were appropriately mapped to the human reference genome (GRCh38) by using HISAT2 (v2.2.1).^[^ ^55^ ^]^ The gene expression quantification was carried out using HTSeq (v2.0.4),^[^ ^56^ ^]^ followed by the DESeq2 (v1.42.0)^[^ ^57^ ^]^ package to obtain DEGs with the criteria of |log2(fold change)| > 1 and Qvalue <0.05. Gene Ontology (GO) biological process term and Kyoto Encyclopedia of Genes and Genomes (KEGG) pathway enrichment analysis of DEGs were performed to identify functional attributes via clusterProfiler (v4.10.0).^[^ ^58^ ^]^

### Immunofluorescence Labeling of Fixed Sample

iPSCs and iPSC‐CMs were plated to Lab‐Tek II chamber slides pretreated with Matrigel (1:250) in DMEM/F12 with L‐glutamine, 15 mm 4‐(2‐Hydroxyethyl)piperazine‐1‐ethanesulfonic acid (HEPES). Cells were fixed with 4% Paraformaldehyde in 1× PBS for 15 min at room temperature (RT) and washed with 1× PBS for two times. Cells were permeabilized with 0.2% Triton X‐100 in PBS for 1 h and blocked with 5% bovine serum albumin (BSA) in 0.1% Triton X‐100 for 2 h at RT. Cells were washed with PBS for two times and subsequently stained with primary antibodies diluted in 1% BSA/0.1% Triton X‐100 in PBS at 4 °C overnight. After two 0.2% Tween 20 in 1× PBS and two 1× PBS washes, cells were incubated with AlexaFluor secondary antibodies at 1:1,000 dilution in 0.1% Triton X‐100/1% BSA at RT for 1.5 h in the dark. Afterward, cells were washed two times with 0.2% Tween 20 in 1× PBS and two times with 1× PBS. Nuclei were stained with Hoechst 33342 trihydrochloride trihydrate at 1:5,000 dilution in 1× PBS for 5 min at RT in the dark, followed by three times 1× PBS washes. For mounting, coverslides were removed from chambers, and ProLong Diamond Antifade mountant (Invitrogen) was added prior to sealing with fingernail polish. For flow cytometry analysis of differentiation efficiency, iPSC‐CMs from all groups were dissociated into single cells with TrypLE Select Enzyme (10X) (Gibco) and then fixed with 4% Paraformaldehyde in 1× PBS for 15 min. Similar to the steps above, cells were then labeled with TNNT2 antibody and Alexa Fluor 488 secondary antibody, as well as Hoechst 33342. Suspension cells were collected by centrifuging at 300 g for 5 min among all treatment steps for medium change. Afterward, the cells were analyzed by flow cytometry on a BD Biosciences FACS Aria II instrument using FACSDiva software. Data analysis was performed using FlowJo X (TreeStar). The antibodies used in the current study are listed in Table S2 (Supporting Information).

### Confocal Imaging and Image Analysis

Immunofluorescence slides and labeled live cell samples were observed using a Nikon 1A confocal microscope. Imaging was performed with Nikon Plan Apo 20×/0.75 DIC, Nikon Plan Fluor 40× oil, 1.30NA, or Nikon Plan Apo OIL 60× oil, 1.40NA objectives (Nikon, Tokyo, Japan). For subsequent data analysis, sarcomere arrangement signals were extracted from each image and subjected to in‐depth analysis through FFT utilizing a customized IDL algorithm. In this process, the digital immunostaining signals of α‐actinin and TNNT2 were transformed from the spatial domain into a frequency‐domain power distribution. The primary peak within this power distribution effectively delineated the principal period pertaining to the α‐actinin and TNNT2 signals along the sarcomere.^[^ ^59^ ^]^ Higher power corresponds to a greater degree of signal distribution regularity and more organized sarcomere structures.

### Calcium Imaging and Data Analysis

For the analysis of spontaneous calcium handling in WT, PLN R9C KI, and patient‐specific iPSC‐CMs, differentiated cells from all groups were seeded on Matrigel‐coated Lab‐Tek II chamber slides and allowed to recover for 3–4 days until normal beating was observed. Cells were loaded with 5 µm of Fluo‐4 AM (Molecular Probe) for 10 min at 37 °C, followed by three thorough washes. The fluorescence signals of Fluo‐4 AM within the beating cells were recorded using a Nikon Ti‐2E microscope platform, customized for high‐content and high‐frame‐rate live cell functional imaging.^[^ ^25^ ^]^ This system was equipped with a SPECTRA III Light Engine solid‐state LED light source (Lumencor, Beaverton, OR) and a Hamamatsu Orca‐fusion Gen‐III sCMOS camera (Hamamatsu, Shizuoka, Japan). The videos capturing calcium dynamics were obtained at a resolution of 2048 × 2048 pixels and a frame rate of 50 frames per second. For ratiometric calcium imaging with Fura‐2 AM, iPSC‐CMs from all groups were seeded at the center of Matrigel‐coated 25 mm coverslips. After recovery for 2–3 days, cells were loaded with 5 µm Fura‐2 AM with 0.1% F‐127 for 10 min at RT in Tyrode's solution (140 mm NaCl, 1 mm MgCl_2_, 5.4 mm KCl, 1.8 mm CaCl_2_, 10 mm glucose, and 10 mm HEPES, pH 7.4). Subsequently, the coverslips were mounted on the imaging platform and subjected to pacing at a frequency of 0.5 Hz (10 volts cm^−1^, bipolar pulse with a 10 ms wave width) within a slotted bath imaging chamber equipped for field stimulation (Warner Instrument LLC, Hamden, CT, Cat#RC‐21BRFS). The Fura‐2 AM signals were captured as ratio pairs using 340/380 nm excitation and 510 nm emission at 2048 × 2048 resolution and a frame rate of 50 ratio pairs per second. The cytosolic calcium signals within individual cells were obtained using Nikon NIS software and further subjected to analysis through customized MATLAB algorithms (MathWorks, Natick, MA).

### Traction Force Microscopy

For the traction force microscopy analysis, the methods were used as reported before.^[^ ^60^ ^]^ The iPSC‐CMs from all the groups were seeded on 96‐well plates at a density of ≈10^5^ cells per well. After recovery and further maturation until day 30 of differentiation, the iPSC‐CMs formed the monolayer structure with >90% of active beating area. The contraction of beating cells was recorded and analyzed using a Sony SI18000 cell motion imaging system. The pixel displacement between each adjacent video frame was calculated, which provided key contractility information such as contractile velocity (µm s^−1^), relaxation velocity (µm s^−1^), percentile of beating area (%), and contraction/relaxation durations (s).

### Transmission Electron Microscopy

The iPSC‐CMs were cultured on Matrigel‐coated tissue culture plates and fixed in 2.5% glutaraldehyde in 100 mm PBS (8 g L^−1^ NaCl, 0.2 g L^−1^ KCl, 1.15 g L^−1^ Na_2_HPO_4_·7H_2_O, 0.2 g L^−1^ KH_2_PO_4_, pH 7.4) overnight at 4 °C. Cells were washed three times with PBS and postfixed in 1% aqueous osmium tetroxide with 1% potassium ferricyanide (FeCN_6_^3−^) for 1 h. Samples were washed three times in PBS and dehydrated through a graded ethanol series (30%–100%), followed by infiltration with Poly/Bed 812 embedding resin (Polysciences). iPSC‐CMs were embedded by inverting Poly/Bed 812‐filled BEEM capsules onto the cultures. Resin blocks were cured overnight at 37 °C and then for 2 days at 65 °C. Monolayers were detached from the culture surface and re‐embedded for cross‐sectioning. Ultrathin sections (≈60 nm) were obtained using a Riechert/Leica UltraCut E ultramicrotome, poststained with 4% uranyl acetate for 10 min and 1% lead citrate for 7 min. Sections were imaged on a JEOL JEM‐1400Flash TEM at 80 kV using a bottom‐mounted AMT digital camera. TEM micrographs were analyzed manually in a blinded manner. At least 20 cells per group were evaluated.

### Data Availability

Detailed experimental methods are available in the Supporting Information. Key research materials are listed in the major resources table in the Supporting Information. The data presented in this study are available on request from the corresponding author. The raw data from RNA‐seq were uploaded to the NIH BioProject database at https://www.ncbi.nlm.nih.gov/bioproject. (ID: PRJNA1272128 and PRJNA1045604)

### Data Plots and Statistical Analysis

All data in this study were presented as mean ± standard error of the mean (SEM) from at least three independent experiments. Statistical analyses were calculated using the unpaired two‐tailed Student's *t*‐test to compare two normally distributed data sets. One‐way or two‐way analysis of variance (ANOVA) tests followed by Holm–Sidak method were used for multigroup comparisons of data. *p* < 0.05 was regarded as significant. The statistical analyses were performed using GraphPad Prism 10.

## Conflict of Interest

SYC has served as a consultant for Merck and United Therapeutics; SYC has held research grants from Bayer and United Therapeutics. SYC is a director, officer, and shareholder of Synhale Therapeutics and Amlysion Therapeutics. SYC has filed patents regarding metabolic dysregulation and inflammation in pulmonary hypertension. The other authors declare that they have no known competing financial interests or personal relationships that could have appeared to influence the work reported in this paper.

## Author Contributions

Q. Y., Y. S., and R. J. B. contributed equally to this work. Conceptualization, HW; Methodology, HW, RJB, QY, KS, QL, and YT; Formal Analysis, HW, YS, QL, XL, and CEB; Investigation, HW, RJB, QY, and QL; Resources, YT, SYC, and JW; Data Curation, HW, YS, and QL; Writing—Original Draft Preparation, HW; Writing—Review and Editing, RJB, SYC, and QL; Visualization, HW, QY, and QL; Supervision, HW and QL; Project Administration, HW and QL; Funding Acquisition, HW, SYC, and QL All authors have read and agreed to the published version of the manuscript.

## Supporting information



Supplemental Table 1

Supplemental Table 3

Supplemental Table 4

Supplemental Figure 1

Supplemental Figure 1

Supplemental Figure 2

Supplemental Figure 3

Supplemental Figure 4

Supplemental Figure 5

Supplemental Figure 6

Supplemental Figure 7

## Data Availability

The data that support the findings of this study are openly available in SRA (Sequence Read Archive) at https://www.ncbi.nlm.nih.gov/sra/PRJNA1272128, reference number 1272128, and https://www.ncbi.nlm.nih.gov/sra/PRJNA1045604, reference number 1045604.
