# Supplementary material for: Promoting Autophagy Mitigates Stress‐Induced Remodeling in Patient iPSC‐CMs with the Phospholamban R9C Mutation
Source: Adv Sci (Weinh). 2025 Nov 27;13(7):e11480. doi: 10.1002/advs.202511480 (PMC12866708; doi:10.1002/advs.202511480)
Supplement: Supplementary file 1 — Supplemental Table 1 [file ADVS-13-e11480-s004.docx]

**Supplemental Table 1. Summary of the sequences of DNA oligos used in the current study.**

| **Gene name** | **Primer sequences** | | **Function** |  |
| --- | --- | --- | --- | --- |
|  | Forward | Reverse |  |  |
| **Target protein** | | | | |
| PLN | CCAGCTAAACACCCGTAAGACT | GAGCGAGTGAGGTATTGGACT | Phospholamban |  |
| **Calcium handling** | | | | |
| ATP2A2 | ATGGGGCTCCAACGAGTTAC | ACACCCACAATTGCATTGGC | ATPase Sarcoplasmic/Endoplasmic Reticulum Ca2+ Transporting 2 |  |
| CASQ2 | AGGAACACCAAAGACCCACTC | GATGCTCAGATCGGGGTTGT | Calsequestrin2 |  |
| RYR2 | CGTGTGGGTCGGAGACATTA | AGAGATTGACAGCCCGATGC | Ryanodine Receptor 2 |  |
| CACNA1C | AGATGACTGCTTATGGGGCTT | CACTGAACCACATGCTTTAGCC | Calcium Voltage-Gated Channel Subunit Alpha1 C |  |
| maSCN5A | CAGCAGTGGAAAGCGGAAC | AGTCCTCTGGGTCCTTGACA | Sodium Voltage-Gated Channel Alpha Subunit 5 |  |
| **Contractile protein** | | | | |
| MYH6 | TGGACAAGCTGCAACTGAAGG | AGTGTCACTCCTCATCGTGCAT | Myosin Heavy Chain 6 |  |
| MYH7 | GCTCTGTGTCTTTCCCTGCT | TGAGGTCAAAAGGCCTGGTC | Myosin Heavy Chain 7 |  |
| MYL2 | CCTTTCCACCATGGCACCTAA | TTTTCACGTTCACTCGCCCA | Myosin Light Chain 2 |  |
| MYL7 | GGGAGAAGCTCAATGGGACAGAC | TGTCAGGGCGAACATCTGCT | Myosin Light Chain 7 |  |
| TNNI1 | CTCCCGCAAACTCTTGCTGA | GGTGTTGTGGAGGCATTTGG | Troponin I1 |  |
| TNNI3 | CAAAGCAAGAGCTGGAGCGA | TCAGATCTGCAATCTCCGTGA | Troponin I3 |  |
| MYBPC3 | CATGAGGCGCGATGAGAAGA | ATGTACTTGCTGCCGCTCAT | Myosin Binding Protein C3 |  |
| TNNT2 | AGACAGAGCGGAAAAGTGGG | GCTGCTTGAACTTCTCCTGC | Troponin T2 |  |
| **Autophagy** | | | | |
| LAMP2 | GGGTTCAGCCTTTCAATGTGAC | GCACAGCATGTCCTGGCT | Lysosomal Associated Membrane Protein 2 |  |
| PINK1 | CGGACGCTGTTCCTCGTTAT | AAATCTGCGATCACCAGCCA | PTEN Induced Kinase 1 |  |
| HSP90B1 | TCTAGGACGGGGAACGACAA | TGGGCTCCTCAACAGTTTCAG | Heat Shock Protein 90 Beta Family Member 1 |  |
| SQSTM1 | GCAACATGGGGCTTGAGAAA | GCCATCGCAGATCACATTGG | Sequestosome 1 |  |
| GPR137 | TCCTCTTCGTGTGGGAGCTA | AGCCTAGACTGCCCGACATA | G Protein-Coupled Receptor 137 |  |
| **Protein degradation ＆ ER stress** | | | | |
| BAG1 | ATGGTTGCCGGGTCATGTTA | CAGCTTGCAAATCCTTGGGC | BAG Cochaperone 1 |  |
| BAG3 | CCCCGTTCAGGTCATCTGTC | TTTCTCGATGGGTCATGGGC | BAG Cochaperone 3 |  |
| CALR | CCCAGAAATTGACAACCCCG | TGCCAAACTCCTCAGCGTAT | Calreticulin |  |
| CALU | AAGCGCTGGATTTACGAGGA | TTGGTGGCAATGAGGTCTCC | Calumenin |  |
| HSPA5 | GTGGAATGACCCGTCTGTGC | GGCTGGTACAGTAACAACTGC | Heat Shock Protein Family A (Hsp70) Member 5 |  |
| HSPA8 | TATTGGAGCCAGGCCTACAC | TCTCGACTTTTCCGTGCTGG | Heat Shock Protein Family A (Hsp70) Member 8 |  |
| HSPA9 | ATGGCACTTCAGAGGGTACG | CCCTTCAAATTGAGCACGGG | Heat Shock Protein Family A (Hsp70) Member 9 |  |
| PRKN | CACCAGCATCTTCCAGCTCA | TCGCCTCCAGTTGCATTCAT | Parkin RBR E3 Ubiquitin Protein Ligase |  |
| DDIT3 | TAAAGATGAGCGGGTGGCAG | GCTTTCAGGTGTGGTGATGT | DNA Damage Inducible Transcript 3 |  |
| HSP90AA1 | GTGGAAGGGCTGTTTCCAGA | CGCCCAGAGTGCTGAATACC | Heat Shock Protein 90 Alpha Family Class A Member 1 |  |
| HSPA1A | AGCTGGAGCAGGTGTGTAAC | CAGCAATCTTGGAAAGGCCC | Heat Shock Protein Family A (Hsp70) Member 1A |  |
| HSPB6 | CGGACGCCTCTTTGACCAG | GTCTAGCAGCACCGAAAAGTG | Heat Shock Protein Family B (Small) Member 6 |  |
| HSPB8 | CAGCAAGAAGGTGGCATTGTT | GGGGAAGCTCGTTGTTGAAA | Heat Shock Protein Family B (Small) Member 8 |  |
| PSMB4 | GGTCATCGGAGGCTATGCTG | TTTTCGGTGACAGTGGCGAT | Proteasome 20S Subunit Beta 4 |  |
| HSPA1B | TGGACTGTTGGGACTCAAGG | AGGGAACGAAACACCCTTAC | Heat Shock Protein Family A (Hsp70) Member 1B |  |
| **Housekeeping gene** | | | | |
| 18S rRNA | CTCAACACGGGAAACCTCAC | CGCTCCACCAACTAAGAACG | 18s Ribosomal RNA |  |

**Summary of sequences of DNA oligos used for PLN Correction.**

| **sgRNA sequence for CRISPR editing** | | |
| --- | --- | --- |
| PLN | TTGAGGCATTTCAATGGTTG |  |
| **ssODN template for PLN R9C mutation correction** | | |
| Correction | GATAAATAGATTCTGTAGCTTTTGACGTGCTTGTTGAGGCATTTCAATGGTTGA**A**GCTCTTCTTATAGCTGAGC**G**AGTGAGGTATTGGACTTTCTCCATG* |  |
| **Genotyping PCR (PCR product is 630 bp)** | | |
| PLN forward | AACAATAGTGCTGAGGAAGATGAA |  |
| PLN reverse | TTTGTGAGCCATGTTGAGGA |  |
| **pMiniT2.0 Sequencing** | | |
| Forward | ACCTGCCAACCAAAGCGAGAAC |  |

* The nucleotide G in underlined bold indicates mutation introduced to the ssODN template for the correction of PLN R9C and, the nucleotide A in bold indicates the conversion of sgRNA PAM region to avoid repeated cutting.

| **gRNA Off Target PCR** | | | | |
| --- | --- | --- | --- | --- |
| Off-target 1 | CTACTTCAGGGGACCGTTCA | TTCATAGGCAAGTGGCAAGA | Chr12 10075479 |  |
| Off-target 2 | GTTGCAGTGAGCCAAGATCA | ATGAGATCCAGGCTGGCTAA | Chr17 30495286 |  |
| Off-target 3 | CAACAGGGACCATGAAGACA | TCCCTCTCCCTGTAAAAGCA | Chr2 198007770 |  |
| Off-target 4 | GGATTGCAGAGCTCTTCCTG | CTGGCTGACATGGTGAAATG | Chr7 27327363 |  |
| Off-target 5 | TGTGCTGCACCCATTAACTC | GGGACCAGGACAGATGAAAA | Chr21 24668849 |  |
