# Supplementary figures and images for: Promoting Autophagy Mitigates Stress‐Induced Remodeling in Patient iPSC‐CMs with the Phospholamban R9C Mutation
Source: Adv Sci (Weinh). 2025 Nov 27;13(7):e11480. doi: 10.1002/advs.202511480 (PMC12866708; doi:10.1002/advs.202511480)

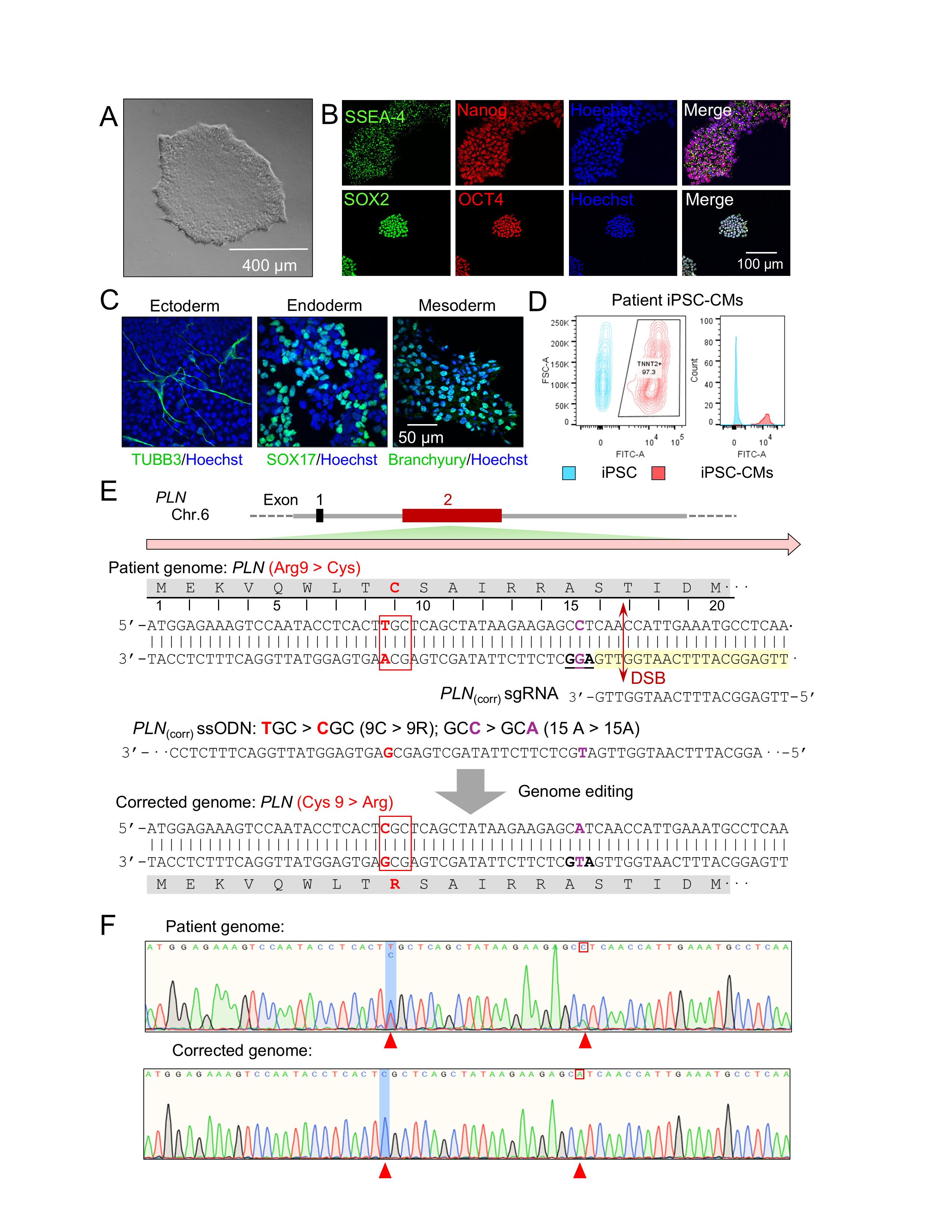

Supplement: Supplementary file 4 — Supplemental Figure 1 [file ADVS-13-e11480-s003.TIF]

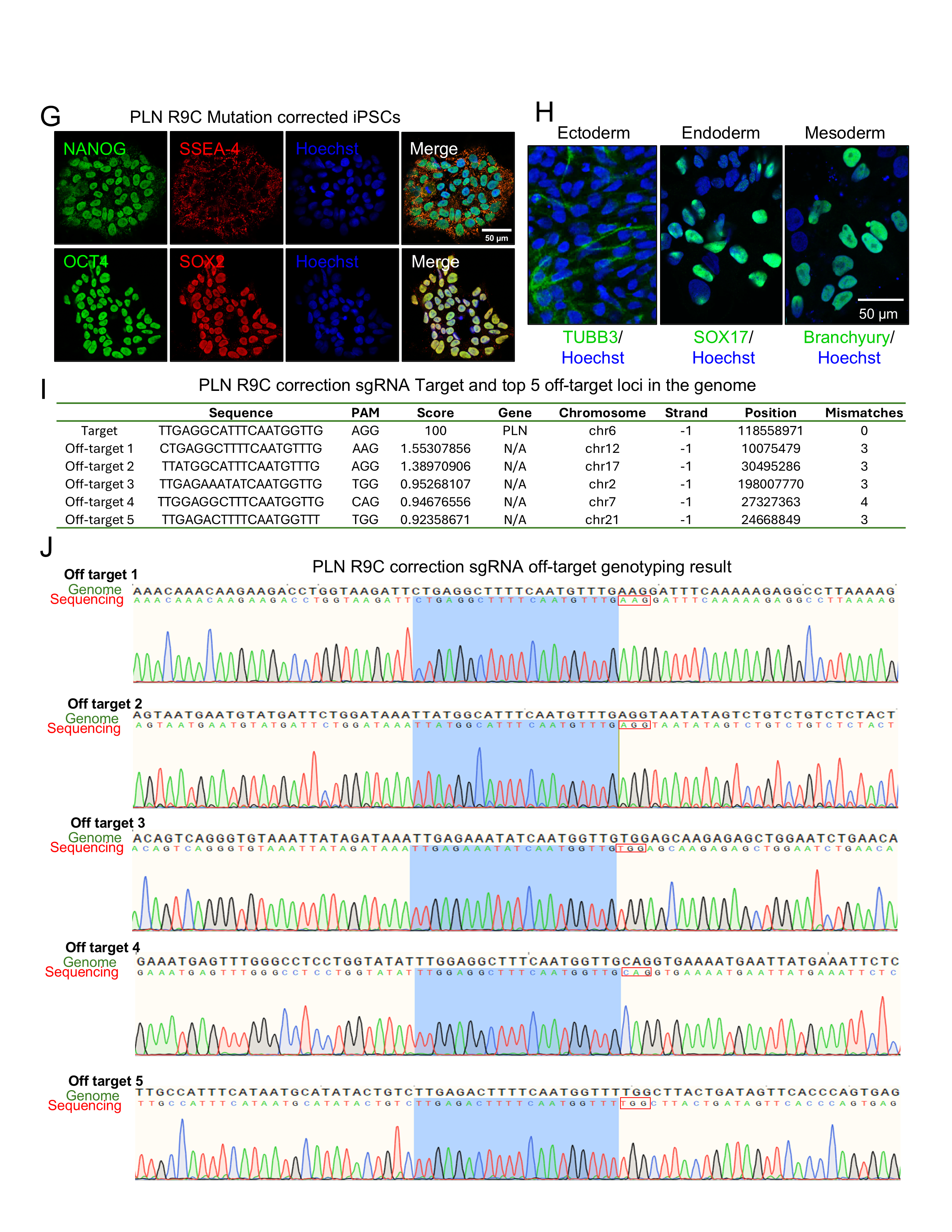

Supplement: Supplementary file 5 — Supplemental Figure 1 [file ADVS-13-e11480-s002.TIF]

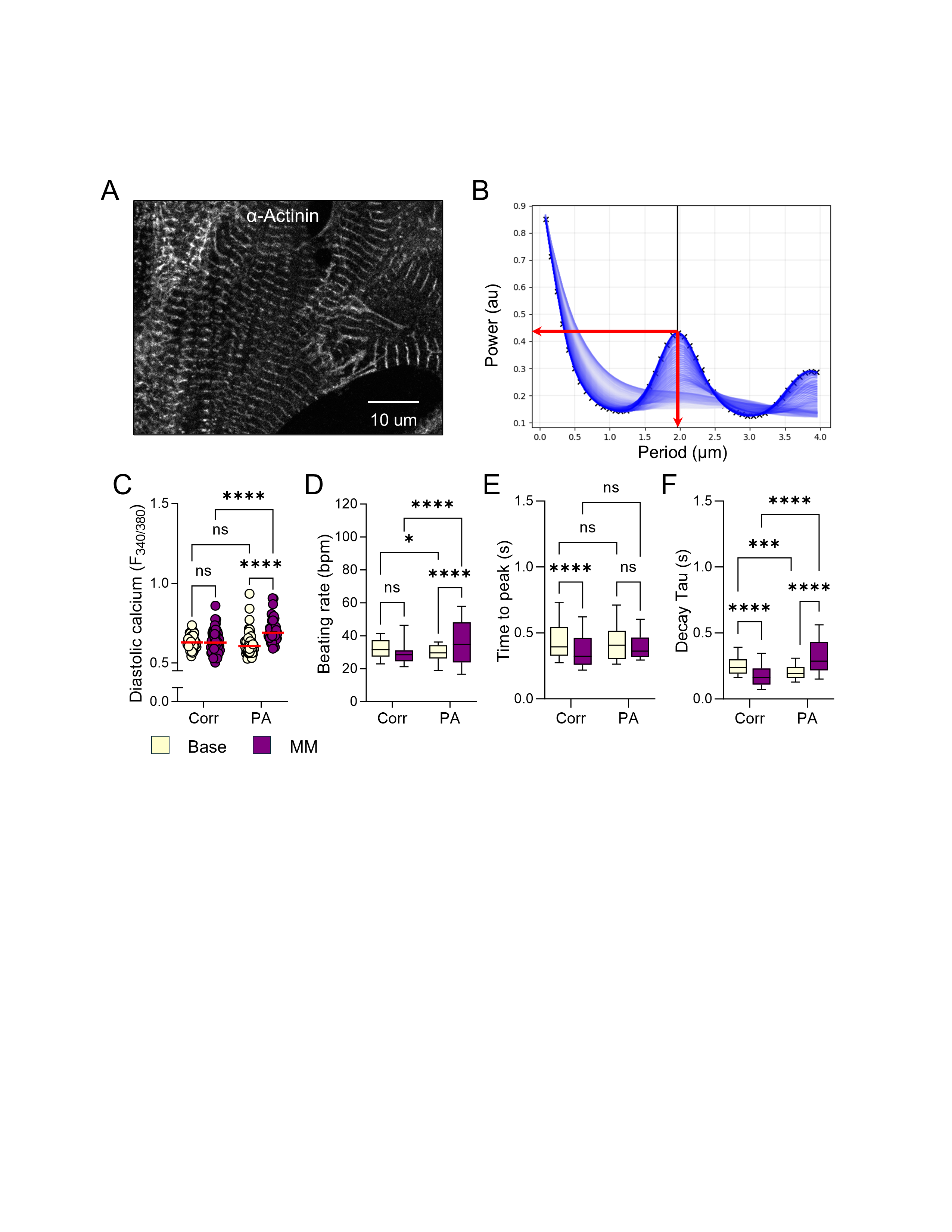

Supplement: Supplementary file 6 — Supplemental Figure 2 [file ADVS-13-e11480-s007.TIF]

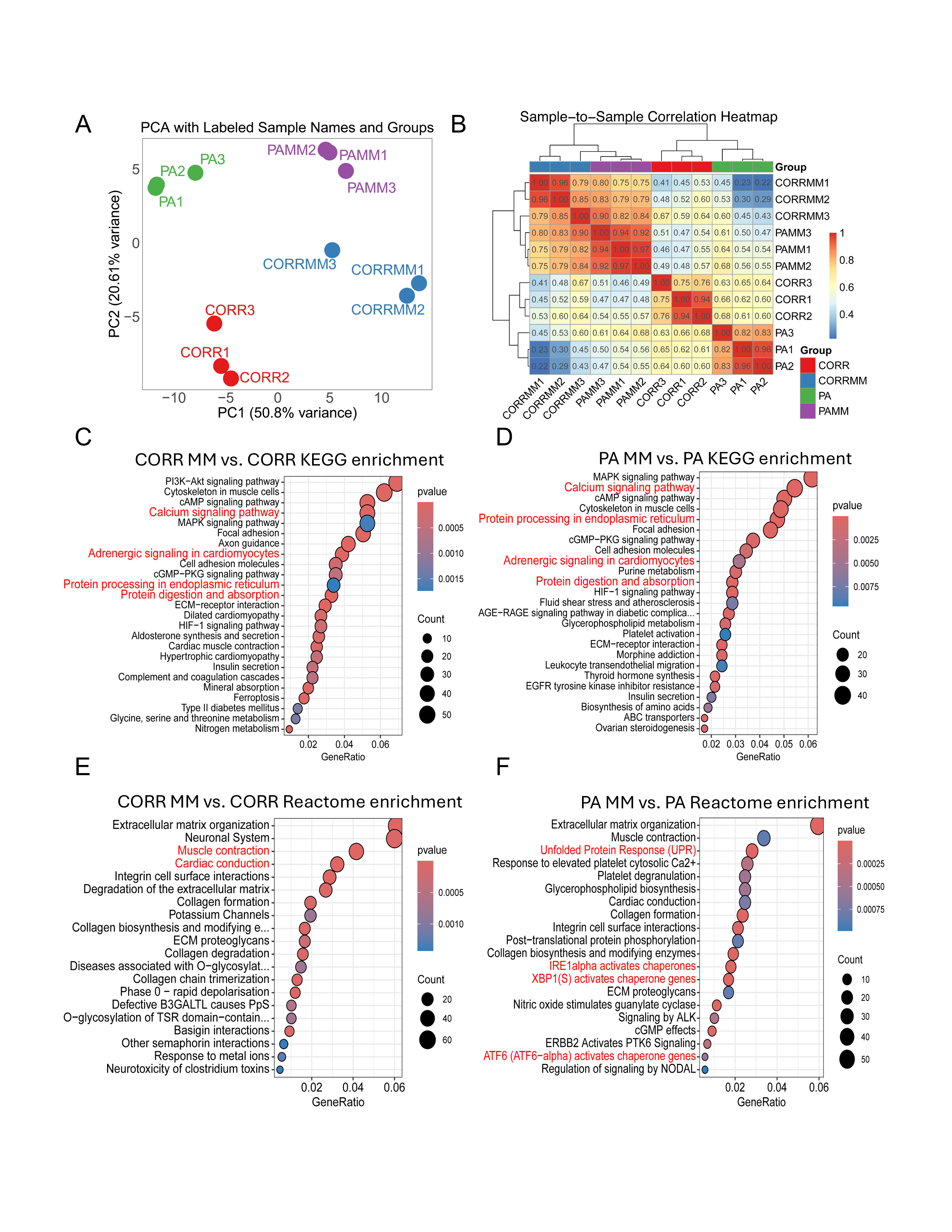

Supplement: Supplementary file 7 — Supplemental Figure 3 [file ADVS-13-e11480-s001.TIF]

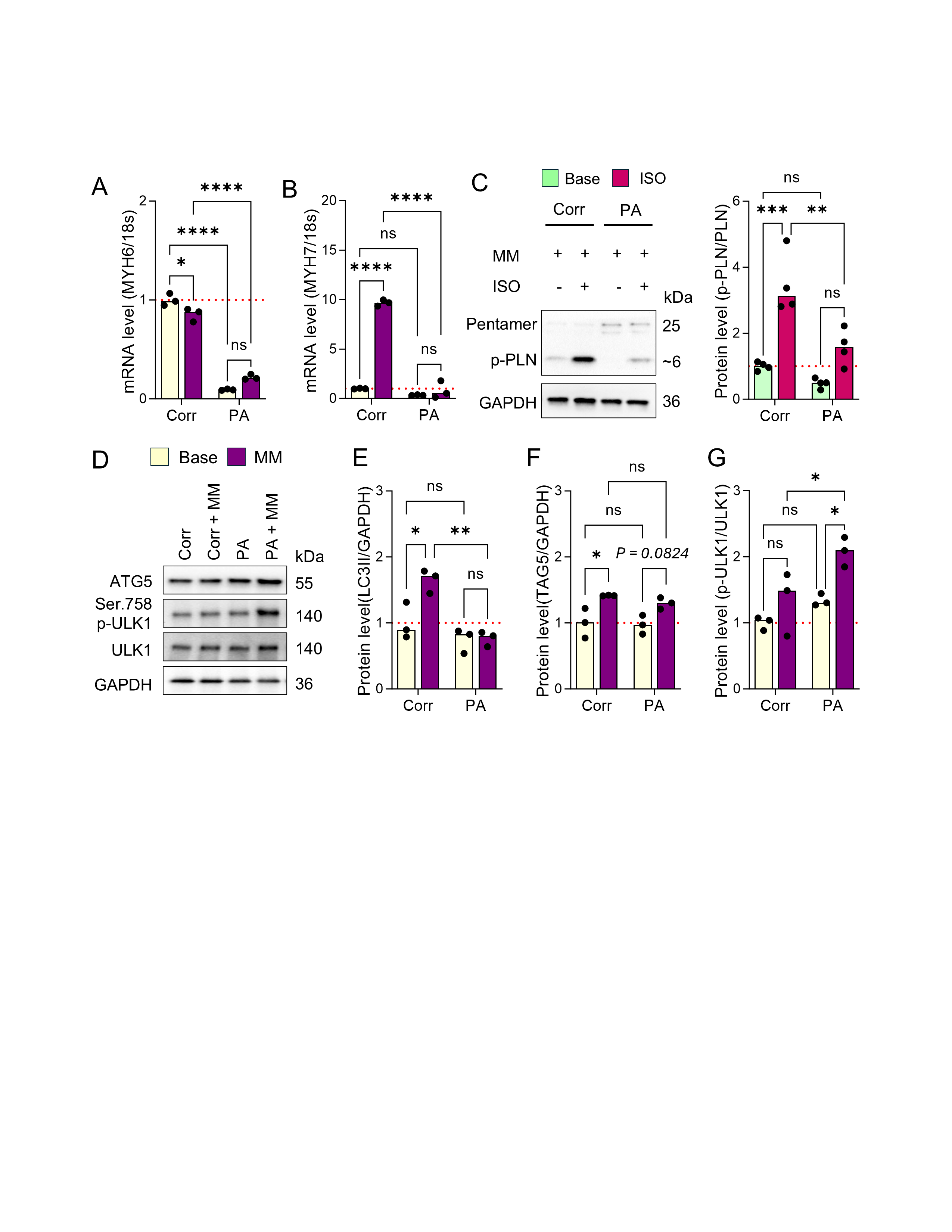

Supplement: Supplementary file 8 — Supplemental Figure 4 [file ADVS-13-e11480-s009.TIF]

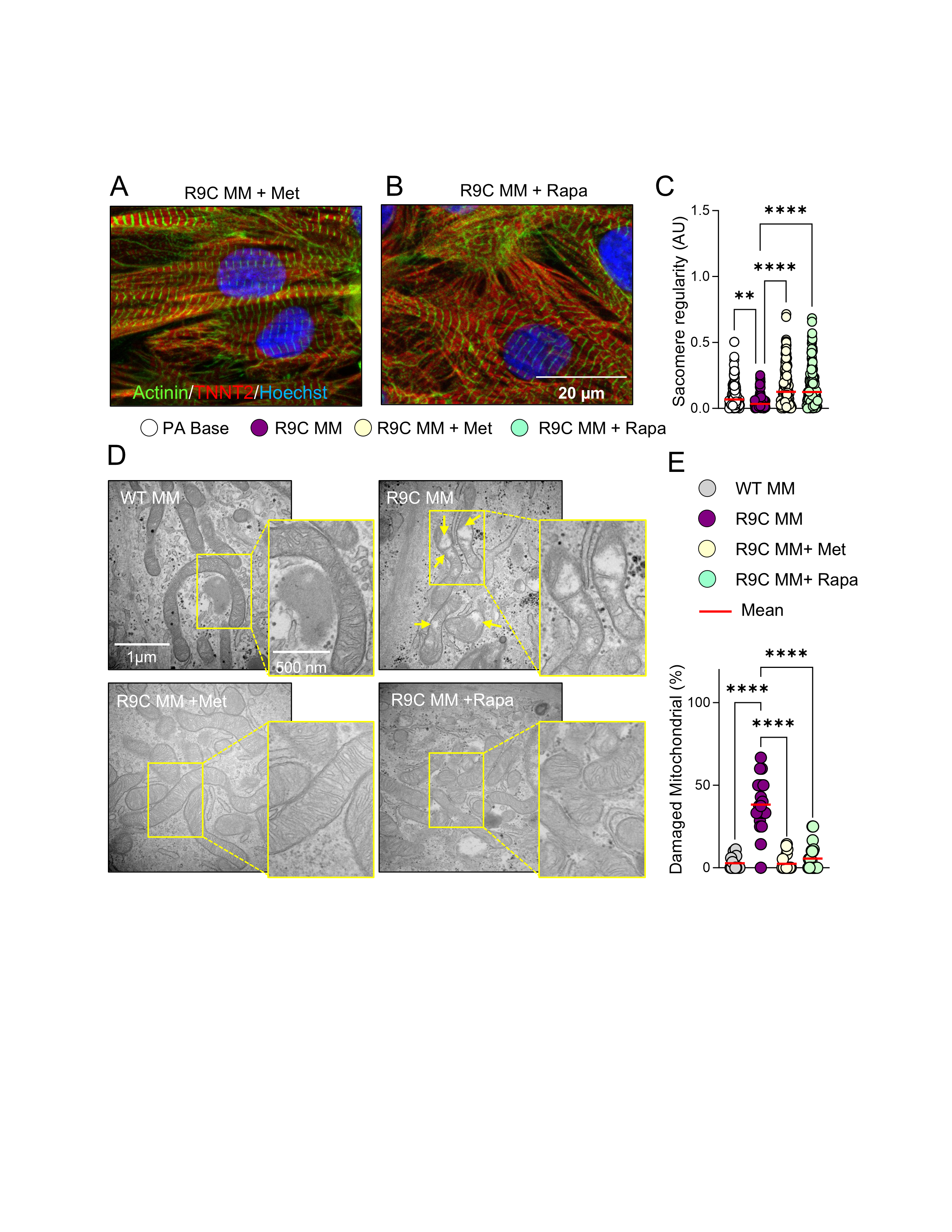

Supplement: Supplementary file 9 — Supplemental Figure 5 [file ADVS-13-e11480-s005.TIF]

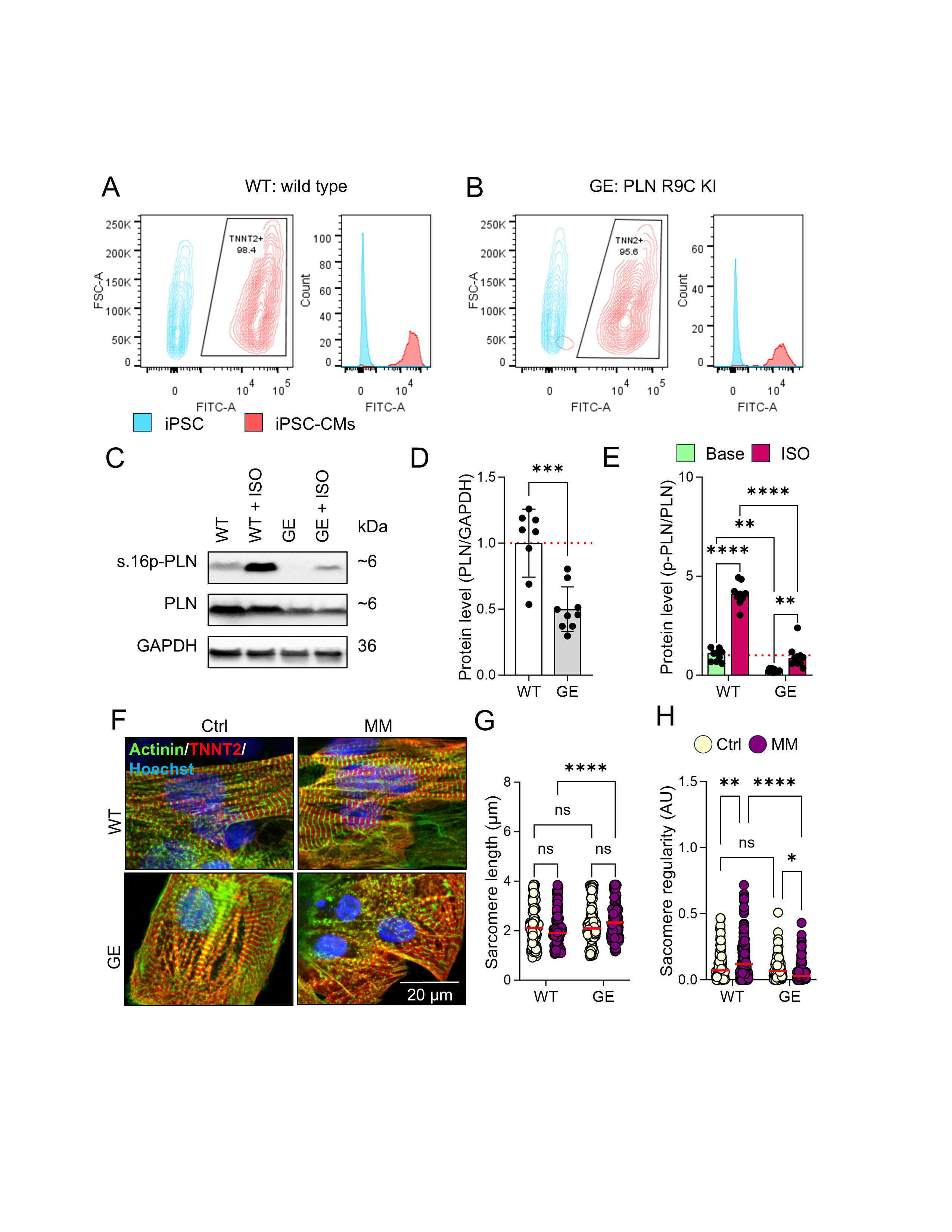

Supplement: Supplementary file 10 — Supplemental Figure 6 [file ADVS-13-e11480-s008.tif]

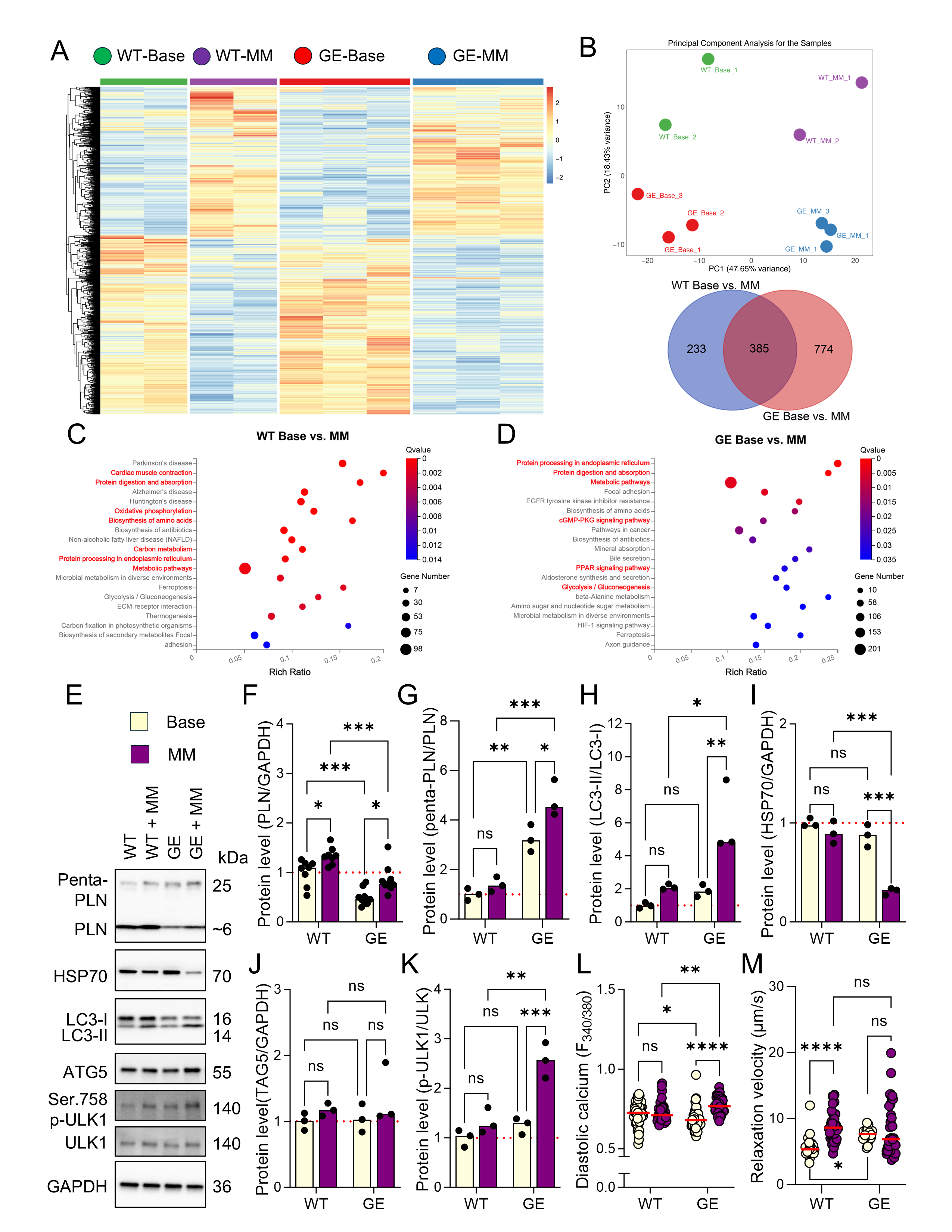

Supplement: Supplementary file 11 — Supplemental Figure 7 [file ADVS-13-e11480-s010.tif]
